# Correlated Vibrational and Electronic Signatures of Surface Disorder in CsPbBr_3_ Nanocrystals

**DOI:** 10.1021/acsnano.5c16045

**Published:** 2025-11-10

**Authors:** Thomas B. Haward, Vincent J.-Y. Lim, Ihor Cherniukh, Maryna I. Bodnarchuk, Maksym V. Kovalenko, Laura M. Herz

**Affiliations:** † Department of Physics, Clarendon Laboratory, 6396 University of Oxford, Oxford OX1 3PU, U.K.; ‡ Department of Chemistry and Applied Biosciences, Laboratory of Inorganic Chemistry, ETH Zürich, Vladimir-Prelog-Weg 1, Zürich CH-8093, Switzerland; § Laboratory for Thin Films and Photovoltaics, Empa−Swiss Federal Laboratories for Materials Science and Technology, Überlandstrasse 129, Dübendorf CH-8600, Switzerland

**Keywords:** lead halide perovskites, nanomaterials, Raman, surface passivation, phonons

## Abstract

Lead halide perovskite nanocrystals have emerged as promising candidates for classical light-emitting devices and single-photon sources, owing to their high photoluminescence quantum yield, narrow emission line width and tunable emission. Judicious choice of ligands to passivate nanocrystal surfaces has proven to be critical to the structural stability and optoelectronic performance of such nanocrystals. While many ligands have been deployed, the resulting quality of the nanocrystal surface can be difficult to assess directly. Here, we demonstrate ultralow frequency Raman spectroscopy as a powerful tool to resolve surface-sensitive changes in size and ligand choice in perovskite nanocrystals. By investigating a size series of CsPbBr_3_ nanocrystals from the strong (5 nm) to the weak (28 nm) confinement range, we show that the line width of Raman-active modes provides a highly selective metric for surface disorder and quality. We further examine a series of 28 nm diameter nanocrystals with four different zwitterionic ligands, unravelling clear links between varying steric effects and surface quality evident from Raman analysis. Photoluminescence and THz photoconductivity probes reveal an evident correlation of charge-carrier dynamics and radiative emission yields with ligand chemistry and surface quality inferred from phonon broadening. We further show that surface defects preferentially trap hot charge carriers, which affects exciton stability and radiative emission yields. Overall, our approach offers powerful insights into optimizing nanocrystal-ligand boundaries to enhance the performance of nanoscale quantum light sources and optoelectronic devices.

## Graphical abstract

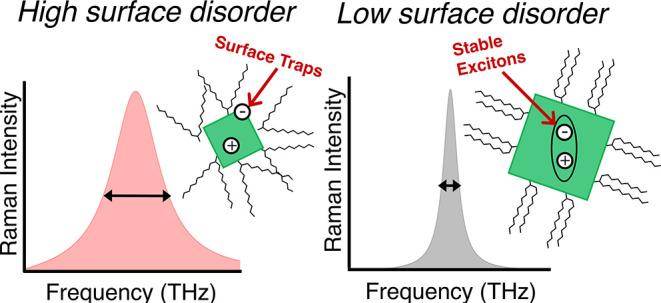

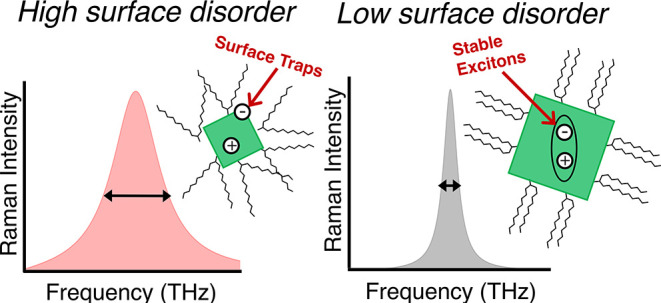

Lead halide perovskite nanocrystals have emerged as promising materials for optoelectronic and quantum photonic applications. The rapid advancements in the performance of colloidal perovskite nanocrystals have been enabled by exceptional intrinsic properties inherited from their bulk counterparts, namely, a large absorption coefficient,^1^ defect tolerance^2^ and high charge-carrier mobility.^3^ Unlike bulk crystals, however, nanocrystals offer spectral tunability through size as well as composition and have exhibited narrow-band emission with near 100% photoluminescence quantum yield (PLQY).^4–6^ These properties have enabled room-temperature single-photon emission, essential for quantum information and computing applications.^7^ Unlike traditional quantum dots such as CdSe, InP and PbS,^8^ perovskite nanocrystals benefit from an intrinsic defect tolerance that enables stable nanocrystal synthesis without the need for an epitaxial core–shell structure.^9^ Instead, lead halide perovskite nanocrystals offer facile passivation by organic ligands that bind to the surface.^10–12^

Essential to the fabrication of single-photon emitters is a route to casting isolated single nanocrystals from a colloid solution. A common approach to achieve such isolation is through sufficient dilution of casting solutions, which, however, requires the surface coverage by ligands to be sufficient and structurally stable.^13,14^ The binding mechanism between surface and ligand is therefore vital to optoelectronic performance, leading to multifaceted design criteria for deployed ligands. Commonly used ligands with firmly bound charged head groups can fill vacancies in the nanocrystal surface, though they have been shown to displace surface ions due to the low internal lattice energy of lead halide perovskites.^15–17^ Large, inflexible ligand tails can be subject to steric hindrance, preventing a complete surface passivation.^18–20^ In contrast, weakly bound ligands are susceptible to dynamic surface migration, leading to ligand desorption under dilution^16,21–23^ and unstable surface reconstruction.^24,25^ Mitigating these effects is thus crucial for achieving stable nanocrystals without compromising optical properties. Judicious choice of ligands is particularly important for very small nanocrystals (≲5 nm), which display strong quantum confinement effects enabling exceptional PLQY,^26–28^ but are susceptible to prominent surface effects resulting from the high surface-area-to-volume ratio.^29^

Given the importance to quantum applications, surface effects in perovskite nanocrystals have thus emerged as a prominent ongoing research area. Recent works have shown postsynthesis treatments,^16,30,31^ the use of zwitterionic ligands^22,32^ and embedding into a solid matrix^33,34^ are able to minimize instability issues. However, the effects of such tuned surface chemistry have to date often been monitored only indirectly.^10,35–38^ Conventional techniques such as PLQY and time-resolved photoluminescence (TRPL) provide insight into optoelectronic quality,^39^ but yield only limited information on the structural and vibrational perturbations induced by inherently defective surfaces.^40^ Obtaining direct information on surface quality is particularly important when nanocrystal size is varied, because the accompanying changes in the degree of quantum confinement will strongly influence photoluminescence efficiency, competing with effects of an increased surface-area-to-volume ratio.^27^ Alternatively, Raman spectroscopy could potentially offer a direct surface-sensitive probe of material structure, yet has up to now been underexploited in the study of nanocrystal surfaces.

In this work, we demonstrate that ultralow frequency Raman spectroscopy is a powerful tool to resolve the surface-sensitive changes in size and ligand choice in perovskite nanocrystals. We show that the line width of Raman-active modes is sensitive to surface disorder and phonon scattering, providing a quantitative metric for surface quality. We further demonstrate clear correlations with optoelectronic quality by deploying several steady-state and time-resolved optical spectroscopic techniques to probe the mobility and recombination dynamics of charge carriers. We observe that improvements in surface quality inferred from surface-dependent vibrational signatures of Raman modes induce enhanced PLQY, suppressed exciton dissociation and lowered surface trapping of charge carriers. Judicious ligand choice thus improves ligand coverage and surface passivation, which in turn reduces nonradiative recombination pathways and phonon damping. We further find that surface traps generated by poor passivation may trap hot carriers, preventing exciton formation and inhibiting optoelectronic performance. Altogether, this work demonstrates a cohesive picture of electronic and vibrational correlations in CsPbBr_3_ nanocrystals, with ultralow frequency Raman spectroscopy emerging as a powerful surface-sensitive technique for probing the surface quality and chemical nature of ligands. This approach offers a clear route to optimizing the performance of optoelectronic and nanoscale photonic devices.

## Results and Discussion

### Fabrication and Characterization of CsPbBr_3_ Nanocrystals

Monodisperse CsPbBr_3_ nanocrystals were synthesized in solution and deposited via drop casting onto z-cut quartz substrates. For the first part of this study, colloidal nanocrystals were prepared with nominal mean edge lengths of 5, 10, 15, 20, and 28 nm, separated by a zwitterionic capping ligand 1,2-dioleyl-SN-glycero-3-phosphoethanolamine (DOPE), while for the second part, 28 nm nanocrystals with a range of different zwitterionic ligands were explored. Full details of the synthesis and deposition procedures are provided in Section 1 of the Supporting Information. Optical and structural characterization of the resultant nanocrystal films is given in Figure 1a. A blueshift in the absorption onset and photoluminescence (PL) is observed with decreasing nanocrystal size as expected, indicative of enhanced quantum confinement.^41^ While nanocrystals of 5 nm edge length are in the strong confinement regime, evidenced by discrete excitonic transition peaks above the absorption onset, the larger nanocrystals transition from the intermediate to the weak confinement regime. We note a somewhat enhanced PL broadening with decreasing nanocrystal size associated with the size distribution across the individual ensembles.^41^ We therefore determined both the mean nanocrystal edge lengths and their distributions from analysis of transmission electron microscopy (TEM) images, shown in Figure 1b for 5, 15, and 28 nm (details of analysis and additional TEM images are shown in the Supporting Information). The TEM micrographs and analysis highlight the high degree of monodispersity and shape-uniformity within cast nanocrystal ensembles.

**Figure 1:**
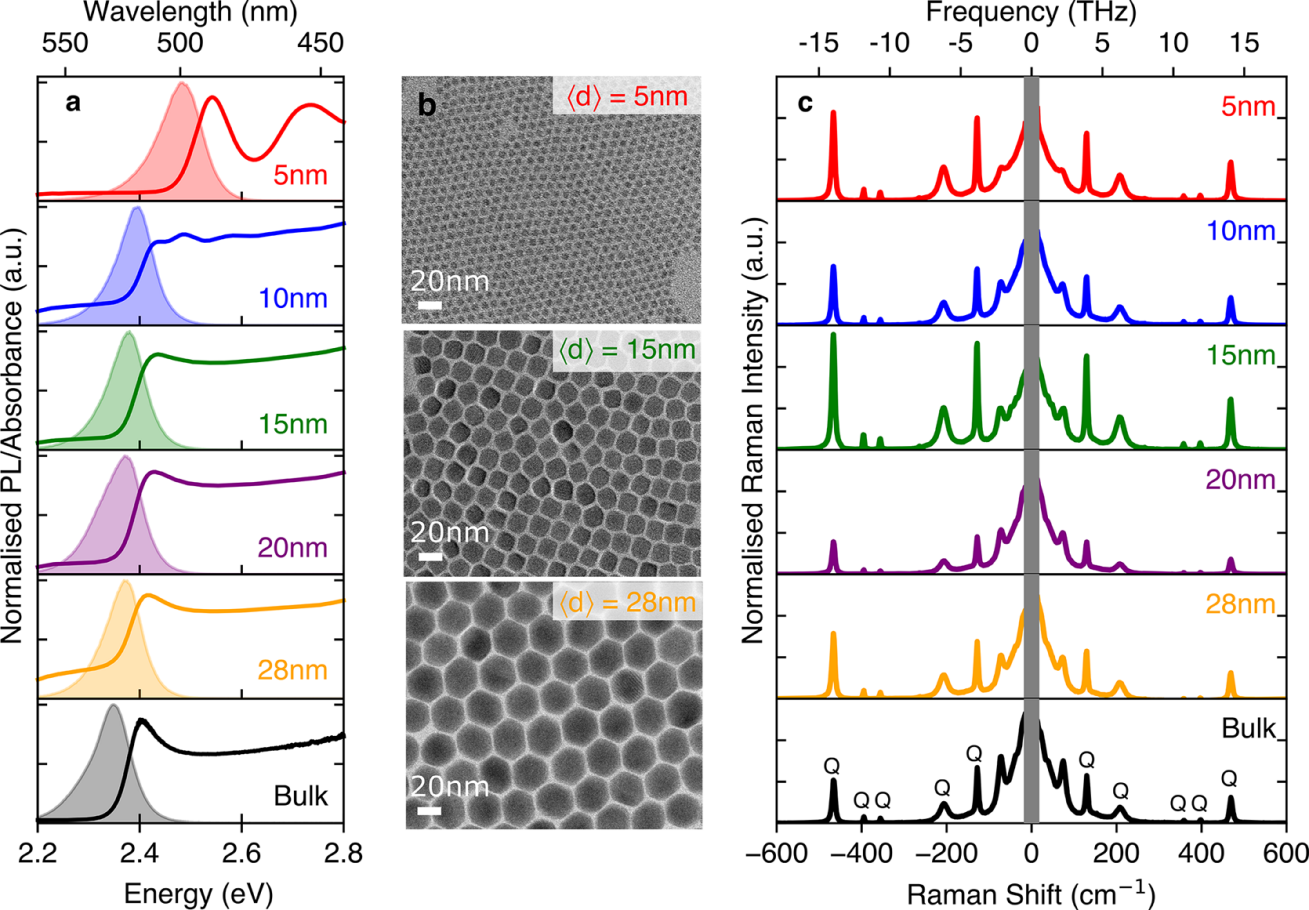
Optical, structural and vibrational characterization of CsPbBr_3_ nanocrystals of varied edge lengths, capped with the DOPE ligand, and of a CsPbBr_3_ bulk thin film. (a) Photoluminescence and absorbance spectra of thin films on quartz substrates. (b) TEM micrographs of monolayer films of three sizes of nanocrystals (further images in Supporting Information). (c) Raman spectra of thin films at terahertz frequencies measured off-resonance with a 900 nm source laser in a backscattering geometry. The Rayleigh scattering peak is indicated in gray. Peaks associated with the quartz substrate are labeled *Q*.

To investigate the influence of surface effects in nanocrystal films, we employed ultralow frequency (ULF) Raman spectroscopy. In order to avoid photoexcitation-induced degradation or overlapping PL signatures, we used a below-gap 900 nm pump to study the off-resonance response of Raman-active phonon modes at terahertz (THz) frequencies. Raman spectroscopy can be a highly surface-sensitive technique because grain boundaries and surfaces break the translational symmetry of a crystal.^42^ In perovskites, in particular, the unsaturated octahedra at the termination site become strained, inducing local disorder. The vibrational response of nanocrystal materials is thus heavily influenced by the disorder at these surfaces.^40,43^ Figure 1c shows the ULF Raman spectra for each of the nanocrystal sizes, in comparison with that for a bulk 3D CsPbBr_3_ thin film. The peaks observed at frequencies >120 cm^–1^ originate from the z-cut quartz substrates (see the quartz-only Raman spectrum in Figure S3). A broad feature rising toward zero frequency is observed for all nanocrystal sizes and the bulk material, which has previously been reported for a wide range of metal halide perovskites,^44–47^ and is commonly referred to as the “central Raman peak” though it comprises a series of peaks centered at low frequencies. Its origin has been intensely debated and ascribed to various factors including local polar fluctuations,^46^ A-site cation rotation^47^ and octahedral tilting from cation lone pairs.^48^ In general terms, such broad central response is indicative of low-energy overdamped Raman-active phonon modes^49^ arising from pronounced lattice anharmonicity present in these soft materials.^50^ The broadening of ultralow energy modes results in a significant vibrational density of states being present even at near-zero frequencies, which are then further amplified in the Raman response through the Bose–Einstein phonon population factor which rises steeply toward zero frequency.^49^ However, individual peaks are still clearly discernible within this broadened response; Raman spectra for all CsPbBr_3_ nanocrystals and the bulk film exhibit a prominent perovskite mode near 74 cm^–1^ that has been attributed to Pb–Br–Pb bond bending in the octahedra.^51,52^ We observe no change in the peak energy of this mode with nanocrystal size, though the peak intensity drops relative to the central mode as the mode broadens in smaller nanocrystals.

### Size-Dependent Analysis of Raman Modes

To explore how such changes in broadening of Raman-active phonon modes are linked with surface effects, we proceed with a quantitative analysis as a function of nanocrystal size. To highlight such effects, Figure 2a shows a narrower range of the same Raman spectra for each nanocrystal size. As done previously, we model Raman modes at terahertz frequencies by a Bose–Einstein population-modified Lorentz model of a damped harmonic oscillator,^45,48,49^ giving a scattering cross section as a function of frequency, *S*(ω), for mode *i* of

1 $$S_{i}=A_{i}\left(n\left(\omega \right)+1\right)\cdot Im\left(\frac{1}{\omega_{i}^{2}-\omega^{2}-i\Gamma_{i}\omega }\right)$$

where *n*(ω) = 1/(*e* ^ℏω/*k*_B_ *T*^ – 1)­is the Bose–Einstein function, *ℏ*, *k*_B_, *T* are the reduced Planck’s constant, Boltzmann constant and temperature respectively, and *A*_*i*_, ω_*i*_, Γ_*i*_ are the harmonic oscillator mode amplitude, frequency and broadening, respectively. We performed fits to the Raman spectra based on a sum of four identified Lorentz oscillator modes associated with CsPbBr_3_, labeled Modes 1–4, plus a small tail associated with a phonon mode of the quartz substrate at 130 cm^–1^. Free parameter fits to eq 1 showed the oscillator frequency, ω_*i*_, to be size-independent for all four identified modes. As such, the oscillators are globally fit to unified values of ω_*i*_ to minimize the degrees of freedom, with variable amplitudes and broadening parameters.

**Figure 2:**
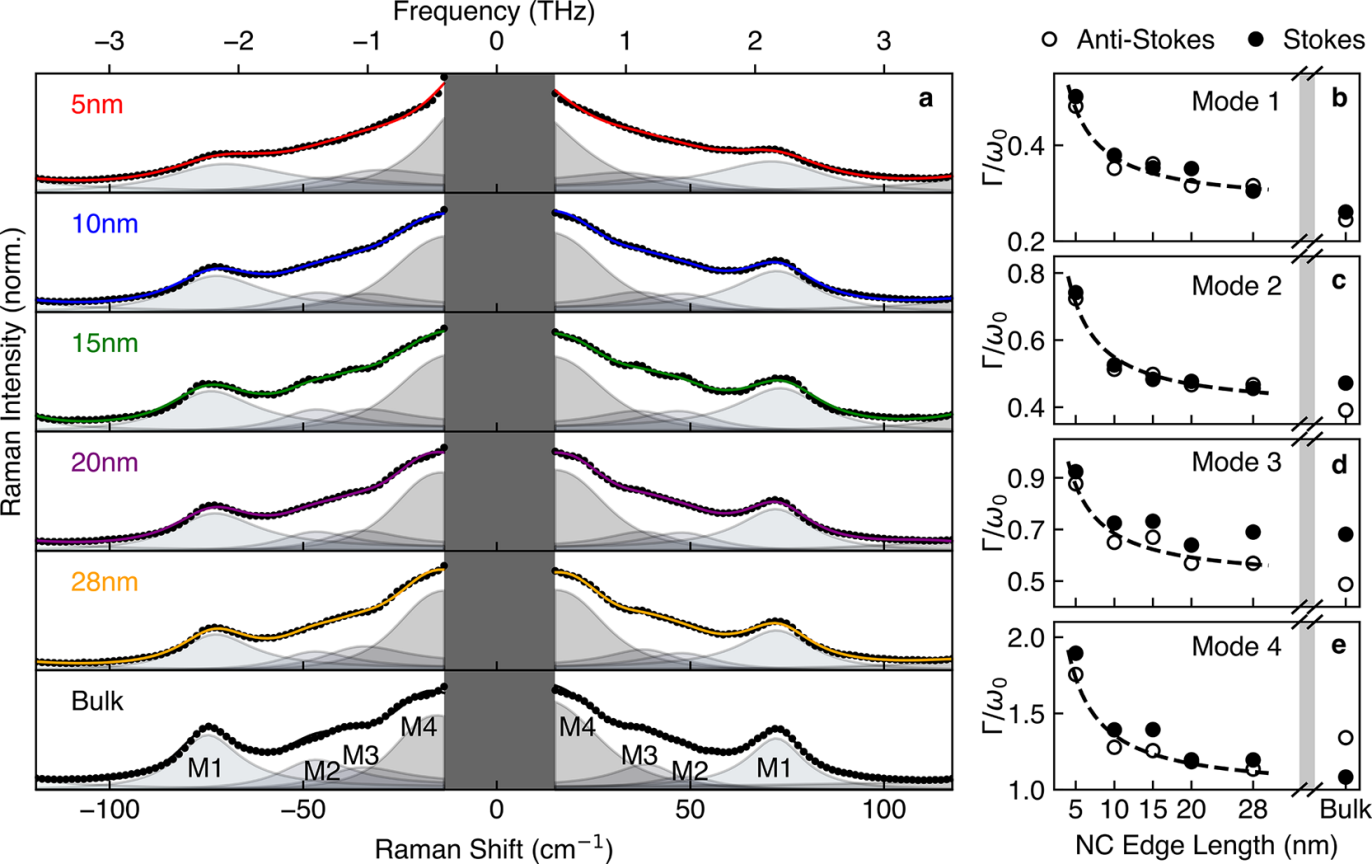
(a) Normalized ultralow frequency Raman spectra (black dots) for CsPbBr_3_ nanocrystals of various edge lengths and for bulk films highlighting the 4 identified phonon modes. The spectra are modeled (colored lines) as a sum of Bose–Einstein-modified Lorentz oscillators, globally fit to the same individual peak frequencies across all sizes. (b–e) The normalized line widths of Modes 1–4 extracted from the model for Stokes (open circles) and anti-Stokes (filled circles) Raman scattering. The dashed line shows the surface-induced broadening model fit to the line width data.

Figure 2b reveals how the normalized line widths Γ_*i*_/ω_*i*_ extracted for the *i*-th mode in eq 1 from such fits depend on the nanocrystal edge length. For all four modes analyzed, a clear trend is observed: with decreasing nanocrystal size, the relative broadening of the Raman-active modes increases, reflecting shorter phonon lifetimes according to the uncertainty relation Γ∼1/τ. We propose that this reduction in phonon lifetime is caused by surface disorder becoming dominant in smaller nanocrystals.^53,54^ The surface-area-to-volume ratio scales inversely with nanocrystal edge length, making contributions from rapid decay mechanisms such as phonon-boundary scattering,^55,56^ phonon-surface defect scattering^57^ and surface strain^58^ far more prevalent in smaller nanocrystals. We quantify this effect through a sum of independent terms arising from intrinsic (bulk-like) and surface-induced broadening contributions, according to

2 $$\Gamma =\Gamma_{0}+\frac{A}{d}$$

where *d* is the nanocrystal edge length, and Γ_0_ and *A* are fit parameters corresponding for each mode to the intrinsic broadening and a proportionality constant for the surface-related broadening, respectively (see Supporting Information Section 4.2 for a full derivation of eq 2). We find that fits of eq 2 to the Raman line width data displayed in Figure 2b well capture the broadening trends with nanocrystal size caused by this additional surface scattering term.

Regarding the interpretation of the observed changes, we note that size-dependent Raman spectra have been reported in the literature for various types of semiconducting nanocrystals.^59–65^ One prominent explanation invoked has been phonon confinement arising from the localization of phonons within the boundaries of the nanocrystal.^61,63^ However, such effects would result in a redshift in phonon energies with decreasing nanocrystal size owing to a relaxation of the Raman selection rules allowing off-center (*q* ≠ 0) phonons to become Raman active.^66,67^ We do not observe such redshifts in the Raman spectra of CsPbBr_3_ nanocrystals (Figure 2) and, as discussed in more detail in Supporting Information Section 4.2, conclude that the nanocrystal edge lengths considered here are too large to exhibit significant optical phonon confinement effects. We further rule out any potential effects arising from variations in the size distribution, as these too should results in a shift in the phonon frequencies with nanocrystal size that is not observed. We therefore instead attribute the size-dependence of the Raman line widths in these nanocrystals to surface effects becoming more dominant as the surface-area-to-volume ratio increases.

### Ligand-Dependent Surface Disorder

We proceed by utilizing the knowledge gained on surface-selectivity of Raman modes to investigate how ligand choice affects the chemical nature of the nanocrystal surfaces. Such organic ligands bind to uncoordinated surface atoms in order to dielectrically separate nanocrystals and passivate their surfaces.^68,69^ We thus expect defect densities and vibrational modes to be influenced by ligand identity, binding strength, and steric configuration. Figure 3 shows the measured Raman spectra for thin films of 28 nm CsPbBr_3_ nanocrystals, prepared with four different organic ligands (optical and structural characterization are provided in Section 5 of the Supporting Information, Figures S4–S7). Each of the four ligands bind to nanocrystal surfaces via a zwitterionic headgroup. Morad et al. have previously shown that the zwitterionic headgroup phosphoethanolamine (PEA) employed here in Ligands 2–4, exhibits an excellent geometric fit to lead bromide nanocrystal surfaces, as the phosphate group coordinates to lead atoms, and the ammonium is inserted into A-site vacancies.^32^ Ligand 1, natural lecithin, provides a mixture of glycerophospholipids with head groups of quaternary and primary ammonium cationic moieties.^32^ It is employed here as a reference ligand owing to its widespread past use in the synthesis of high-quality CsPbBr_3_ nanocrystals.^23,25,70–73^ However, because of the mixture of bulky glycerophospholipids, electrostatic and steric effects between lecithin molecules prevent complete surface coverage.^25^ Ligand 1 has recently been outperformed by the geometrically optimized zwitterionic ligands (Ligands 2–4) that theoretically offer 100% coverage.^32^ We further note that the phosphoethanolamine head groups deployed in Ligands 2–4 contain only a primary ammonium cation. Differences in optoelectronic and vibrational properties between these three ligands are therefore attributed solely to the influence of their different long-chain tails (see molecular structures in Figure 3).^54,74^ The tails of Ligand 2 are highly unsaturated, long alkyl chains, which provide a durable surface coating. However, it has been shown for 2D layered perovskite materials that unsaturated bonds have limited flexibility, causing steric hindrance that dictates a less dense surface coverage and induces surface strain.^75,76^ Ligand 3 has shorter, saturated and symmetric tails, allowing tighter packing, better coverage and a low surface energy.^19,77,78^ The single tail of Ligand 4 is a polyethylene glycol (PEG) chain whose repeating C–O–C units provide degrees of freedom inducing flexibility for the ligand to adapt to the geometric configuration of its surroundings,^79,80^ minimizing steric effects between neighboring ligands and improving nanocrystal surface coverage. These molecules are widely used as surface passivants, including in 3D perovskite-based devices^81^ and in traditional semiconductor nanocrystals,^82,83^ though have only recently been adopted in the surface passivation of lead halide perovskite nanocrystals.^32,84,85^

**Figure 3:**
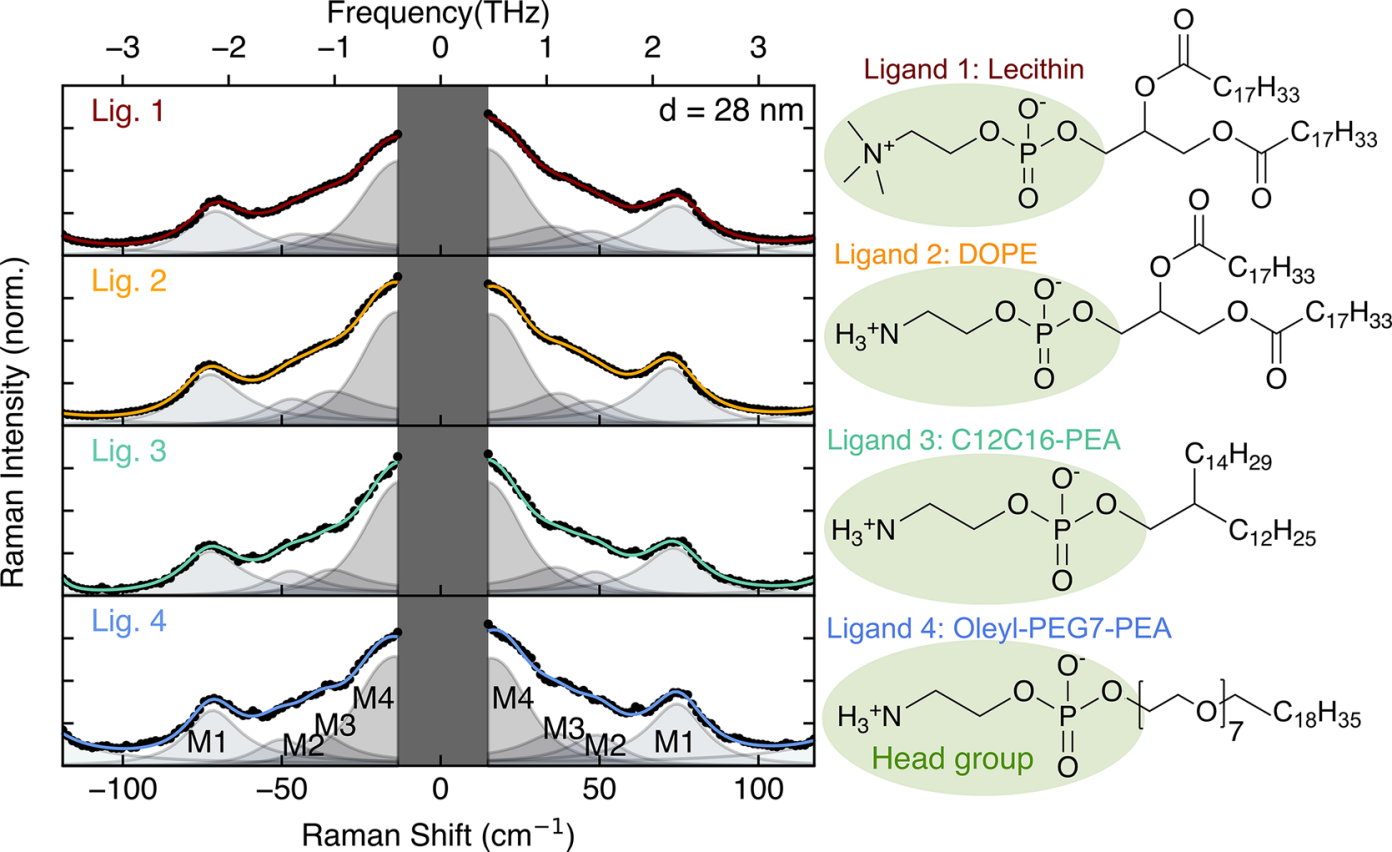
Normalized ultralow frequency Raman spectra of 28 nm CsPbBr_3_ nanocrystals capped with four different zwitterionic ligands (black dots). The Raman spectra are modeled (colored lines) as a sum of four Bose–Einstein-modified Lorentz oscillators with central frequencies at the identified Raman modes. Ligands 2–4 share the same phosphoethanolamine headgroup, though the tails of all four ligands differ in their chemical composition, length, and saturation.

### Optoelectronic Evidence of Surface Disorder

In order to probe the dependence of ligand-induced surface passivation, the Raman spectra shown in Figure 3 were modeled, again using eq 1. The extracted values for the normalized line width of the most prominent Raman mode (M1) at 74 cm^–1^ reveal a clear variation with ligand type that is consistent with the qualitative understanding of their chemical characteristics. These changes, displayed in Figure 4a, are more subtle than the variation in Raman line widths observed in the size-dependent spectra, but follow a similar rationale. A similar systematic variation of the normalized line width is also observed for Modes 2 and 3, shown in Figure S8. While the surface-area-to-volume ratio remains unchanged for these 28 nm diameter nanocrystals, the choice of different ligands results in surface effects that broaden phonon modes when the nanocrystal-ligand interface is strained. Ligand 1 yields the broadest mode indicating enhanced phonon scattering and surface disorder, likely because of the poor surface coverage of the glycerophospholipid mixture. In contrast, the narrowest mode is found for Ligand 4, evidencing the improved surface passivation and stability enabled by the phosphoethylene glycol chain and phosphoethanolamine headgroup.

**Figure 4:**
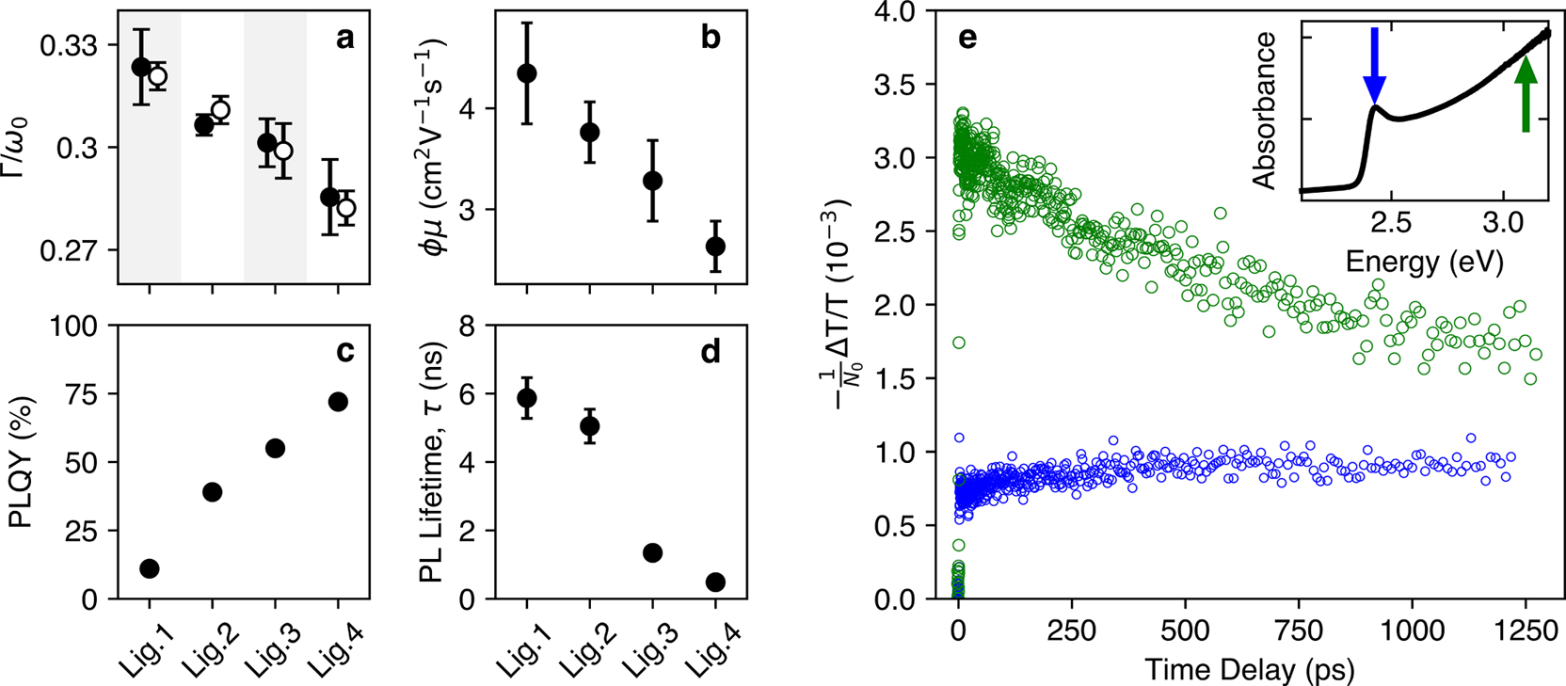
Raman and optoelectronic properties of 28 nm nanocrystals terminated with the four different ligands displayed in Figure 3. For each ligand, in the same order, subpanels (a–d) display: (a) the normalized line width of the Stokes (open circles) and anti-Stokes (filled circles) Raman M1 mode at 74 cm^–1^ extracted from fits to Raman spectra shown in Figure 3, shifted relative to one another to allow for easier visualization; (b) the effective terahertz electron–hole sum mobility obtained from OPTP transients on deposited films; (c) PLQY values measured in solution; and (d) fluence-independent PL lifetimes extracted from time-resolved PL measurements. (e) OPTP photoconductivity transients recorded for the film of 28 nm nanocrystals terminated with Ligand 1, following excitation with an optical pulse at photon energy just above the absorption onset (2.41 eV, blue), and high in the bands (3.1 eV, green), normalized by the initially injected charge-carrier density. Owing to the low signal for 2.41 eV excitation, each time delay point was integrated over 400,000 pulses, versus 100,000 pulses for 3.1 eV excitation. The inset shows the absorbance spectrum of 28 nm nanocrystals capped with lecithin, highlighting the energetic location of the two pump energies.

We proceed by correlating the surface effects revealed through the Raman probe with key optoelectronic markers of nanocrystal quality. For this purpose, we employ several optical spectroscopy techniques that provide critical performance indicators, namely photoluminescence quantum yield (PLQY) spectroscopy in the steady-state, as well as time-resolved photoluminescence (TRPL) and THz photoconductivity. The resulting metrics, displayed in Figure 4b–d, reveal a coherent dependence on the ligand surface effects uncovered by the Raman probes. As discussed in detail below, these combined probes identify surface-mediated charge-carrier trapping processes as a key hindrance to the performance of nanocrystal films, which can however be addressed by judicious ligand choice.

We begin with analysis of the trends in the PLQY recorded for nanocrystals prepared in solution with each ligand, shown in Figure 4c. Nanocrystal solutions were chosen because their PLQY reflects intrinsic emission quality, while in solid-state films, extrinsic effects such as poor photon outcoupling and surface scattering, often dominate. The trend in PLQY with ligand choice mirrors that of the Raman line widths in Figure 4a: Ligand 1 has the largest phonon line width and lowest PLQY, while Ligand 4 exhibits a much narrower line width with the highest emission efficiency. This anticorrelation indicates that improved surface passivation increases the proportion of radiative versus trap-mediated recombination of charge carriers. As the head groups of Ligands 2–4 are identical, we propose that the observed differences arise from enhanced surface coverage suppressing nonradiative recombination centers at the surfaces of nanocrystals. This is consistent with previous reports of both traditional^86–88^ and perovskite^21,89,90^ nanocrystals, in which surface disorder is understood to introduce surface trap states and suppress radiative recombination.

We proceed by examining the correlation with PL lifetimes extracted from time-resolved PL (shown in Figure S9 in the Supporting Information). As Figure 4d reveals, somewhat counterintuitively, Ligand 4 exhibits both the shortest PL lifetime and the highest PLQY, and this inverse trend persists across Ligands 1–3. PL transients show no significant differences in decay dynamics with changes in excitation fluence (Figure S9), which, together with the very high PLQY observed, suggests that monomolecular recombination of excitons dominates the PL decay. CsPbBr_3_ nanocrystals have been shown to exhibit very fast nanosecond exciton lifetimes,^91,92^ owing to a high oscillator strength, as spatial confinement induces significant electron–hole wave function overlap, even in weakly confined nanocrystals.^13,93–95^ Suppressing this rapid radiative exciton recombination would therefore prolong the PL lifetime. We attribute the variation in PL lifetime to the surface trap states capturing single electrons (or holes), effectively dissociating an exciton and leaving the corresponding holes (or electrons) within the bulk of the material. This process, caused by poor passivation of the surfaces, both reduces the PLQY and prolongs the PL lifetime through spatial separation of electrons and holes, consistent with the anticorrelation of PLQY and PL lifetimes displayed in Figure 4c,d.

To investigate such exciton dissociation by surface trap states and probe the trapping dynamics, we carried out optical-pump terahertz-probe (OPTP) spectroscopy. Here, the nanocrystal film is initially excited with an optical pulse and probed at a varied time delay by a terahertz pulse with subpicosecond resolution. The terahertz probe is sensitive only to charged mobile species, such as free electrons or holes, and it is not resonant with interexcitonic transitions and is thus “blind” to any excitonic species. Following photoexcitation, a finite population of free charge carriers may be generated inside the nanocrystal, leading to photoconductivity transients such as those displayed in Figures 4e and S10 in the Supporting Information. As such, the initially generated photoconductivity will therefore depend sensitively on both the mobility μ of free charge carriers and the absorbed-photon-to-free charge branching ratio ϕ. Femtosecond THz-frequency pulses have been shown to probe only intrananocrystal charge-carrier motion and transport, and so are ideal for observing changes in boundary scattering and charge-carrier trapping without the influence of internanocrystal transport.^41,96^ Figure 4b shows the product of the two quantities, the effective electron–hole sum mobility (ϕμ), derived from photoconductivity values recorded immediately following 3.1 eV excitation (a full description of the method is provided in Section 6.3 of the Supporting Information). Given that these values are recorded for one particular nanocrystal size (28 nm edge length) but differ appreciably for the use of different capping ligands, such variations can be attributed to factors altering the branching between the free charge carrier and exciton populations (reflected in the values of ϕ). In particular, the dissociation of excitons resulting from trapping of electrons (holes) by surface defects leaves free holes (electrons) in the core of the nanocrystal that enhance ϕ and therefore the extracted effective mobility values ϕμ. We find that nanocrystals with Ligand 1, exhibiting the lowest PLQY and longest PL lifetime, exhibit the highest ϕμ value. Again, a clear trend is found through to Ligand 4, which exhibits the lowest surface strain, highest PLQY, fastest excitonic PL lifetime and lowest ϕμ, in accordance with the most effectively suppressed surface trapping and therefore enhanced exciton stability.

### The Role of Hot Carrier Trapping

We further show that such charge trapping at nanocrystal surfaces preferentially affects charge carriers generated with significant excess energy (hot carriers). Following excitation with 3.1 eV photons, the photoconductivity peaks within the <1 ps system response before decaying (see Figures 4e and S10), as is typically observed for instantaneous free carrier generation in bulk materials.^97^ At this photon energy the pump pulse initially generates hot carriers high in the conduction band (see inset in Figure 4e) that thermalize to the band edge within ∼100 fs,^38,98,99^ followed by cooling and population decay. The fast generation of a free-carrier response in these excitonic systems thus suggests that hot carriers are rapidly trapped by surface states in a process that competes with thermalization and exciton formation. Indeed, Ye et al. showed a correlation between defect density in perovskite nanocrystals and the rate of hot carrier cooling, attributing faster cooling to an enhanced hot carrier trapping mechanism.^2^ To investigate such effects further, we measured OPTP photoconductivity transients for nanocrystals capped with Ligand 1, both with significantly above-gap excitation at 3.1 eV, and with 2.41 eV excitation at the band edge (Figure 4e). We would expect that band edge excitation generates bound excitons with small excess energy that require little thermalization and cooling and so do not exhibit hot-carrier trapping. Indeed, the initial photoconductivity value immediately following this resonant excitation is significantly lower than when excitation significantly above gap occurs, as the ultrafast (subpicosecond) hot-carrier trapping mechanism is now suppressed. Instead, following 2.41 eV excitation, a slow rise in photoconductivity is observed over hundreds of picoseconds, indicating that cold carriers are trapped by unsaturated surface trap states at a much lower rate. In contrast, excitation at 3.1 eV with high excess energy excitation results in ultrafast trapping of hot carriers within the carrier cooling time scale, both generating a high photoconductivity signal and saturating surface traps such that slower cold carrier trapping becomes negligible. As shown in Figure S11, this stark difference in photoconductivity at different excitation energies is observed at multiple fluences. This observation mirrors measurements by Righetto et al.,^100^ who showed that the PLQY of MAPbBr_3_ nanocrystals decreased monotonically with excess excitation energy, which they also attributed to surface trapping of hot carriers. Overall, the strong correlations between PLQY, PL lifetimes, free-charge generation and Raman probes of surface quality clearly demonstrate how ligand optimization, from Ligand 1 through to Ligand 4, can enable high-quality passivation of nanocrystal surfaces, suppressing hot-carrier trapping, enabling radiative excitonic recombination, and therefore boosting optoelectronic performance. Our approach further highlights the predictive power of Raman spectroscopy to provide a quantitative metric for the quality of ligands deployed in the surface passivation of lead halide perovskite nanocrystals.

## Conclusion

In conclusion, we have demonstrated the critical importance of nanocrystal surfaces on both the vibrational and optoelectronic properties of CsPbBr_3_ nanocrystal films. We have employed ultralow frequency Raman spectroscopy to directly probe the effects of increasing surface-area-to-volume ratio with decreasing nanocrystal size, without the need to distinguish the effects of electronic quantum confinement. Through comparative analysis of zwitterionic ligands with varying steric effects, we revealed a cohesive correlation between ligand chemistry, phonon broadening, charge-carrier dynamics and radiative emission yields. The observed trends demonstrate the power of ligand optimization, with more complete ligand coverage suppressing surface defects capable of trapping hot charge-carriers, thus boosting exciton stability and enhancing radiative recombination rates. Similarly, strongly confined nanocrystals with a high surface-area-to-volume ratio exhibit more significant surface effects than their larger counterparts, evidenced by phonon mode broadening that scales inversely with size. The magnitude of surface effects was found to correlate with a increase in PL lifetime and an increase in effective charge-carrier mobility, as the exciton population is diminished by surface defects that selectively trap one type of charge carrier. Our use of multimodal optical spectroscopy techniques thus enables a broader understanding of correlations between optoelectronic performance of CsPbBr_3_ nanocrystals, and surface quality derived from Raman spectroscopy which emerges as a powerful and accessible tool to quantify such effects. Ultimately, this study provides fundamental insights into the optimization of nanocrystal-ligand boundaries for improving the performance of nanoscale quantum light sources and optoelectronic devices.

## Methods and Experiments

### Nanocrystal Fabrication and Deposition

Colloidal nanocrystals were grown and capped by ligands in solution. The films were deposited by drop casting 14 μL of NCs solution (∼32 mg/mL, in hexane/octane 9:1, v/v) onto z-cut quartz substrates. Further details are provided in Section 1 of the Supporting Information.

### Absorption Measurements

Transmittance and reflectance were measured using a Fourier Transform Infrared (FTIR) Spectrometer (Bruker Vertex 80v), relative to a reference z-cut quartz substrate. A tungsten halogen lamp was used as the source with a silicon detector, and CaF_2_ beamsplitter.

### Photoluminescence

Steady-state photoluminescence spectra were measured using an intensified charge-coupled device (iCCD). Samples were excited by a 398 nm diode laser (Picoquant LDH-D-C-398M). The photoluminescence was dispersed by a grating spectrometer (Princeton Instruments SP-2558) and recorded on a silicon iCCD (Princeton Instruments PI-MAX4).

### Time-Resolved Photoluminescence

Time-resolved photoluminescence (TRPL) was measured using time-correlated single-photon counting (TCSPC). Samples were excited using a 398 nm picosecond pulsed laser diode at a repetition rate of 1 MHz. The photoluminescence signal was dispersed by a grating spectrometer (Princeton Instruments SP-2558) and detected using a single-photon avalanche detector. Transients were recorded as histograms, as events were binned using a PicoHarp300 TCSPC event timer.

### Optical-Pump Terahertz-Probe Spectroscopy

Optical-pump terahertz-probe (OPTP) measurements were performed using an amplified Ti:sapphire femtosecond laser (Spectra-Physics Spitfire). The amplifier generates an 800 nm output with a 5 kHz repetition rate and 35 fs pulse duration. This fundamental beam was used to generate the 400 nm pump beam via a beta-barium-borate (BBO) crystal and the 515 nm pump beam via an optical parametric amplifier, single-cycle terahertz pulses via a spintronic emitter and a gate beam for detection via electro-optic sampling. A delay stage was used to vary the optical path difference between pump and probe beams.

### Transmission Electron Microscopy

Monolayer nanocrystal films were prepared on carbon-coated copper TEM grids. Transmission electron microscopy images were collected using a JEOL JEM2200FS microscope operating at 200 kV accelerating voltage.

### Photoluminescence Quantum Yield

The photoluminescence quantum yield of the CsPbBr_3_ nanocrystals in solution was measured in a Hamamatsu Quantaurus-QY Plus UV–NIR absolute photoluminescence spectrometer (C13534-11) equipped with an integrating sphere.

### Ultralow Frequency Raman Spectroscopy

A Spectra Physics Matisse 2 TS Ti:sapphire continuous wave (CW) laser pumped with a Spectra Physics Millennia 532 nm CW pump laser was used as the excitation source, operating at 900 nm with a 50 kHz spectral line width. The beam was focused onto thin film samples using a 0.5 NA microscope objective (Olympus LMPLFLN50x) and the scattered radiation collected in a backscattering geometry. Raman spectra were measured using a Horiba iHR320 spectrometer and a Symphony silicon CCD. OptiGrate Bragg filters were used to spectrally narrow the incident laser and remove Rayleigh scattering from the sample.

## Supplementary Material



## Notes

Fabrication methods, experimental details, the calculation of effective terahertz electron–hole sum mobility, and the derivation of the size-dependent Raman broadening equation, as well as additional data including TEM images, X-ray diffraction patterns, absorbance spectra, steady-state PL spectra, Raman spectra, film thicknesses via surface profilometry, and fluence-dependent TRPL and OPTP transients (PDF)
