# Supplementary material for: Correlated Vibrational and Electronic Signatures of Surface Disorder in CsPbBr3 Nanocrystals
Source: ACS Nano. 2025 Nov 10;19(46):40159–69. doi: 10.1021/acsnano.5c16045 (PMC12659429; doi:10.1021/acsnano.5c16045)
Supplement: Supplementary file 1 [file nn5c16045_si_001.pdf]

SUPPORTING INFORMATION: Correlated vibrational and electronic signatures of surface disorder in $CsPbBr_3$ nanocrystals

*Thomas B. Haward[1], Vincent J.-Y. Lim[1], Ihor Cherniukh[2], Maryna I. Bodnarchuk[2], Maksym V. Kovalenko[2], Laura M. Herz[1]**

1 - Department of Physics, Clarendon Laboratory, University of Oxford, Oxford OX1 3PU, U.K.

2 - ETH Zürich, Department of Chemistry and Applied Biosciences, Laboratory of Inorganic Chemistry, Vladimir-Prelog-Weg 1, Zürich CH-8093, Switzerland; Empa–Swiss Federal Laboratories for Materials Science and Technology, Laboratory for Thin Films and Photovoltaics, Überlandstrasse 129, Dübendorf CH-8600, Switzerland

1 SAMPLE FABRICATION 4

1.1 Nanocrystals of Varied Size 4

1.2 Nanocrystals with Varied Ligands 6

2 EXPERIMENTAL METHODS 8

2.1 Photoluminescence 8

2.2 Linear Absorption Spectroscopy 8

2.3 Transmission Electron Microscopy (TEM) 8

2.4 X-Ray Diffraction 8

2.5 Ultra-low frequency Raman spectroscopy 9

2.6 Photoluminescence Quantum Yield (PLQY) 10

2.7 Time-resolved Photoluminescence 10

2.8 Optical-Pump Terahertz-Probe Spectroscopy 10

3.1 Transmission Electron Microscopy 12

3.2 X-Ray Diffraction 13

4 RAMAN ANALYSIS 14

4.1 Identifying Substrate Modes 14

4.2 Modelling Nanocrystal Size-Dependent Raman Modes 14

5 LIGAND-DEPENDENT CHARACTERIZATION 17

5.1 Steady State Optical Spectroscopy 17

5.3 Transmission Electron Microscopy 19

5.4 X-Ray Diffraction 20

5.5 Film Thickness 21

5.6 Ligand-Dependent Linewidths of Raman Modes 22

6 TIME-RESOLVED SPECTROSCOPY 24

6.1 Time-Resolved Photoluminescence 24

6.2 Fluence-Dependent Optical-Pump Terahertz Probe (OPTP) Spectroscopy **26**

6.3 Calculating the Effective Charge-Carrier Mobility **28**

1 Sample Fabrication

1.1 Nanocrystals of Varied Size

Ligand: 1,2-Dioleoyl-SN-Glycero-3-Phosphoethanolamine

Chemicals

Cs_2CO_3 (99.9%, Sigma-Aldrich); Bis(2,4,4-trimethylpentyl)phosphinic acid (TMPPA, 90%, Fluorochem); $PbBr_2$ (99.999%, Aldrich); trioctylphosphine oxide (TOPO, 90%, Strem); n-octane (97%, Acros Organics); hexane (≥95%, Sigma-Aldrich); Oleic acid (OA, 90%, Sigma-Aldrich); 1,2-Dioleoyl-sn-glycero-3-phosphoethanolamine (DOPE, >98%, Apollo Scientific); mesitylene (99%, Thermo Scientific Chemicals).

Stock solutions

$PbBr_2$-TOPO stock solution was prepared by dissolving 367 mg $PbBr_2$ and 2.15 g TOPO in 5 mL n-octane at 120 °C, followed by cooling down, dilution with 20 mL hexane, and filtering over a 0.22 µm PTFE filter. Cs-TMPPA stock solution was prepared by reacting 100 mg Cs_2CO_3 with 1 mL TMPPA in 2 mL n-octane at 100 °C, followed by cooling down, dilution with 27 mL hexane, and filtering over a 0.22 µm PTFE filter. 0.1 M DOPE stock solution was prepared by dissolving 74.4 mg of DOPE in 1 mL of toluene.

Synthesis of the nanocrystals was done by modifying the reported method[1]

<u>Synthesis of 5.4 nm $CsPbBr_3$ nanocrystals:</u> 13 mL of hexane was mixed with 1 mL of $PbBr_2$ stock solution in a 25-mL flask. Next, under heavy stirring, 0.5 mL of Cs-TMPPA stock solution was injected. After 5 min of NC growth, 0.1 mL of 0.1 M DOPE solution was added to the crude solution. In 2 min after the addition of ligand, the crude solution was concentrated by evaporating hexane on a rotary evaporator. To purify nanocrystals, 1.5 mL of acetone was added to ∼1.6 mL

of the concentrated crude solution, followed by centrifugation and solubilization of the obtained nanocrystals in 1 mL hexane. The nanocrystals were precipitated once more with 0.7 mL of acetone, centrifuged and redissolved in 0.5 mL of hexane, followed by addition of 10 μL of 0.01 M DOPE.

Synthesis of 10.4 nm $CsPbBr_3$ nanocrystals: 3.5 mL of hexane was mixed with 3 mL of $PbBr_2$ stock solution in a 25-mL flask. Next, under heavy stirring, 1.5 mL of Cs-TMPPA stock solution was injected. After 4 min of NC growth, 0.15 mL of 0.1 M DOPE solution was added to the crude solution. In 2 min after the addition of ligand, the crude solution was concentrated by evaporating hexane on a rotary evaporator. To purify nanocrystals, 1 mL of acetone was added to ~2 mL of the concentrated crude solution, followed by centrifugation and solubilization of the obtained NCs in 1 mL hexane. The nanocrystals were precipitated once more with 1 mL of acetone, centrifuged and redissolved in 1.5 mL of hexane, followed by addition of 30 μL of 0.01 M DOPE.

Synthesis of 15.3–28.4 nm $CsPbBr_3$ nanocrystals: The nanocrystals were synthesized by a modified $PbBr_2$-TOPO approach[1] with a slow injection of Cs and $PbBr_2$ stock solutions at a higher temperature. At the end of injection, the nanocrystals were capped with DOPE ligand, washed twice with acetone (1:0.5–1 by volume), and redispersed in hexane.

Film deposition

The quartz substrates were cleaned by sequentially sonicating at 50 °C in Hellmanex (2% in water), distilled water, isopropanol, and acetone for 10 minutes at each stage. The films were deposited by drop-casting 14 μL of nanocrystals solution (~32 mg/mL, in hexane:octane 9:1, v/v) onto the substrates.

Monolayer nanocrystal films for transmission electron microscopy (TEM) imaging were prepared by drop-casting 10 μL of ~2 mg/mL nanocrystal solution in hexane onto carbon-coated copper TEM grids (Ted Pella #01801) placed on a paper tissue. The protective formvar layer was removed by immersing the grids in toluene for ~10 s prior to film deposition.

1.2 Nanocrystals with Varied Ligands

Chemicals

Cs_2CO_3 (99.9%, Sigma-Aldrich); $PbBr_2$ (99.999%, Aldrich); trioctylphosphine oxide (TOPO, 90%, Strem); hexane (≥95%, Sigma-Aldrich); 1,2-, mesitylene (99%, Thermo Scientific Chemicals); Lecithin for biochemistry (>=97%, Roth AG); phosphorus(V) oxychloride (99%, Sigma-Aldrich); 2-aminoethan-1-ol (≥99.0%, Sigma-Aldrich); Oleic acid (OA, 90%, Sigma-Aldrich); acetic acid (>99.8%, Sigma-Aldrich); triethylamine (99%, Sigma-Aldrich); 2-dodecylhexadecan-1-ol (>93%, TCI); Polyethylene glycol monooleyl ether, n=7 (Oleyl-PEG7, TCI).

<u>Synthesis of ligands</u>: 2-Ammonioethyl (oleylpolyethyleneglycolyl, n = 7) phosphate (Oleyl-PEG7-PEA) ligand was synthesized as reported in ref.[2]. Solution of Oleyl-PEG7 (0.0125 mol; 1 equiv; 7.175 g) dissolved in dry tetrahydrofuran (THF) (12.5 mL) along with triethylamine (0.01375 mol; 1.1 equiv; 1.915 mL) is added dropwise with vigorous stirring to a solution of phosphorous oxychloride (0.015 mol; 1.2 equiv; 1.39 mL) in THF (1.25 mL) on an ice water bath. The reaction mixture is subsequently kept at 20 °C for 20 min to complete the reaction. Next, ethanolamine (0.015 mol; 1.2 equiv; 0.905 mL) and triethylamine (0.03 mol; 2.4 equiv; 4.18 mL) in THF (18.75 mL) are added dropwise under vigorous stirring to the reaction mixture kept in a room-temperature water bath. Subsequently, the mixture is heated to 40 °C for 20 min to complete

the ring closure. Finally, the reaction mixture is filtered to remove precipitated triethylamine hydrochloride, and the filtrate solution is dried. The product is further purified from triethylamine hydrochloride by centrifuging three times with diethyl ether, followed by drying on rotary evaporator. An oily residue, that is, alkyl-2-oxo-1,2,3-oxazaphospholane, is dissolved in a mixture of acetic acid (2.85 mL) and distilled water (1.3 mL) at 70 °C. After 30 min, ring scission at the P–N bond is complete, and the product is beaten with cold acetone and left in the freezer for two days. The product (1 g) is precipitated, then washed with cold acetone and dried. A 0.05 M ligand stock solution was prepared by dissolving 69.9 mg of Oleyl-PEG7-PEA in 2 mL of toluene. 2-Ammonioethyl 2-dodecyl-1-hexadecyl phosphate (C12C16-PEA) ligand was synthesized as reported in ref.[2] and dissolved in mesitylene to form 0.1 M solution. 0.13 M Lecithin stock solution was prepared by dissolving 50 mg of lecithin in 1 mL of anhydrous toluene.

Synthesis of the nanocrystals: The nanocrystals were synthesized by modifying the reported method[1] with a slow injection of Cs and $PbBr_2$ stock solutions at a higher temperature. At the end of injection, the nanocrystals were capped with either C12C16-PEA, or lecithin, or Oleyl-PEG7-PEA ligands, washed twice with acetone (1:0.5–1 by volume), and redispersed in hexane.

Film deposition

The quartz substrates were cleaned by sequentially sonicating at 50 °C in Hellmanex (2% in water), distilled water, isopropanol, and acetone for 10 minutes at each stage. The films were deposited by drop casting 14 μL of nanocrystals solution (~32 mg/mL, in hexane:octane 9:1, v/v).

2 Experimental Methods

2.1 Photoluminescence

Steady-state photoluminescence spectra were measured using an intensified charge-coupled device (iCCD). Samples were excited by a 398nm diode laser (Picoquant LDH-D-C-398M). The photoluminescence was dispersed by a grating spectrometer (Princeton Instruments SP-2558) and recorded on a silicon iCCD (Princeton Instruments PI-MAX4).

2.2 Linear Absorption Spectroscopy

Transmittance (T) and reflectance (R) spectra were measured using a Fourier Transform Infrared (FTIR) Spectrometer (Bruker Vertex 80v), relative to a reference z-cut quartz substrate. A tungsten halogen lamp was used as the source with a silicon detector, and CaF_2 beamsplitter. Absorbance, A, was calculated from T and R as

$$A = -\ln\left(\frac{T}{1-R}\right) \tag{S1}$$

2.3 Transmission Electron Microscopy (TEM)

Monolayer nanocrystal films were prepared on carbon-coated copper TEM grids. Transmission electron microscopy images were collected using a JEOL JEM2200FS microscope operating at 200-kV accelerating voltage.

2.4 X-Ray Diffraction

X-ray diffraction (XRD) patterns were measured using a PANalytical X'pert powder diffractometer, with a Cu K-α_1 radiation source. The scans were in the range 5-45 ^{o}, at a scan speed of 0.01 $^{o}s^{-1}$ with a 0.004 ^{o} step size. The diffraction patterns were adjusted by the z-cut quartz substrate peak at 16.43^{o} to account for sample tilt.

2.5 Ultra-low frequency Raman spectroscopy

Raman spectroscopy in the ultra-low frequency terahertz region was performed using a home-built system. A Spectra Physics Matisse 2 TS Ti:Sapphire continuous wave (CW) laser pumped with a Spectra Physics Millennia 532 nm CW pump laser was used as the excitation source, operating at 900nm with a 50kHz spectral linewidth. The laser spectrum was narrowed, and spontaneous emission removed by two band pass filters. The light was normally incident on the sample and collected via a back-scattering geometry. A 0.5 NA microscope objective (Olympus LMPLFLN50x) focused the light onto the sample and collimated the back-scattered radiation. A beamsplitter was used to reflect elastic (Rayleigh) scattering and transmit Raman shifted radiation. Two additional notch filters were used to remove elastic scattering and transmit inelastic scattering. The light then passed through a spatial filter and was spectrally dispersed by a Horiba iHR320 spectrometer using a 600 g/mm, 750 nm central wavelength grating. An N_2-cooled Symphony silicon CCD was used as the detector. The bandpass filters, beamsplitter and notch filters were volume Bragg filters from OptiGrate, designed to operate at the 900 nm laser wavelength. The FWHM linewidth of these filters is 7 cm^{-1} and the resolution varied between samples depending on surface quality, thickness and alignment, so the lowest accessible Raman shift is taken as 10 cm^{-1}. The recorded spectra underwent three corrections. Firstly, a background spectrum measured under the same conditions but without a sample was subtracted from the sample spectrum. Secondly, the Si CCD was calibrated using a tungsten-filament reference lamp of known emissivity to account for the detector's spectral response. Finally, the Raman intensity is proportional to the fourth power of frequency/Raman shift, which was corrected for in all recorded spectra.

2.6 Photoluminescence Quantum Yield (PLQY)

The photoluminescence quantum yield of the $CsPbBr_3$ nanocrystals in solution was measured in a Hamamatsu Quantaurus-QY Plus UV–NIR absolute photoluminescence spectrometer (C13534-11) equipped with an integrating sphere.

2.7 Time-resolved Photoluminescence

Time-resolved photoluminescence (TRPL) was measured using time-correlated single-photon counting (TCSPC). Samples were excited using a 398nm picosecond pulsed laser diode at a repetition rate of 1 MHz. The photoluminescence signal was dispersed by a grating spectrometer (Princeton Instruments SP-2558) and detected using a single-photon avalanche detector. Transients were recorded as histograms, as events were binned using a PicoHarp300 TCSPC event timer.

2.8 Optical-Pump Terahertz-Probe Spectroscopy

Optical-pump terahertz-probe (OPTP) measurements were performed using an amplified Ti:Sapphire femtosecond laser (Spectra-Physics Spitfire). The amplifier generates an 800 nm output with a 5 kHz repetition rate and 35 fs pulse duration. This fundamental beam is split to generate pump and probe beams. Firstly single-cycle THz pulses are generated via the inverse spin Hall effect by a spintronic emitter ($W/Co_{40}Fe_{40}B_{20}/Pt$) multilayer film on quartz. Secondly, 400 nm optical pump pulses are generated via second-harmonic generation in a beta-barium-borate (BBO) crystal. Fractional changes in THz transmission are detected via free-space electro-optic sampling using a 1 mm-thick (110)-ZnTe crystal in the frequency range 0.5-2.5 THz. Delay stages are used to vary the delay between pump and probe pulses on a fs-ps timescale. THz emission and detection optics, including the sample, are kept under a vacuum of pressure <0.1 mbar throughout

the experiment. For measurements with a 2.41 eV (515 nm) pump, the central wavelength of the 400nm beam was converted using an optical parametric amplifier (Light Conversion, TOPAS-C). Comparisons between 2.41 (515 nm) eV and 3.1 eV (400 nm) were performed at a fluence of 35.5 μJcm^{-2}. The THz response of a material is quantified as the photoinduced change in the THz transmission, thereby accounting for the absorption of THz radiation by phonons in the dark.

3 Size-dependent Structural Characterization

3.1 Transmission Electron Microscopy

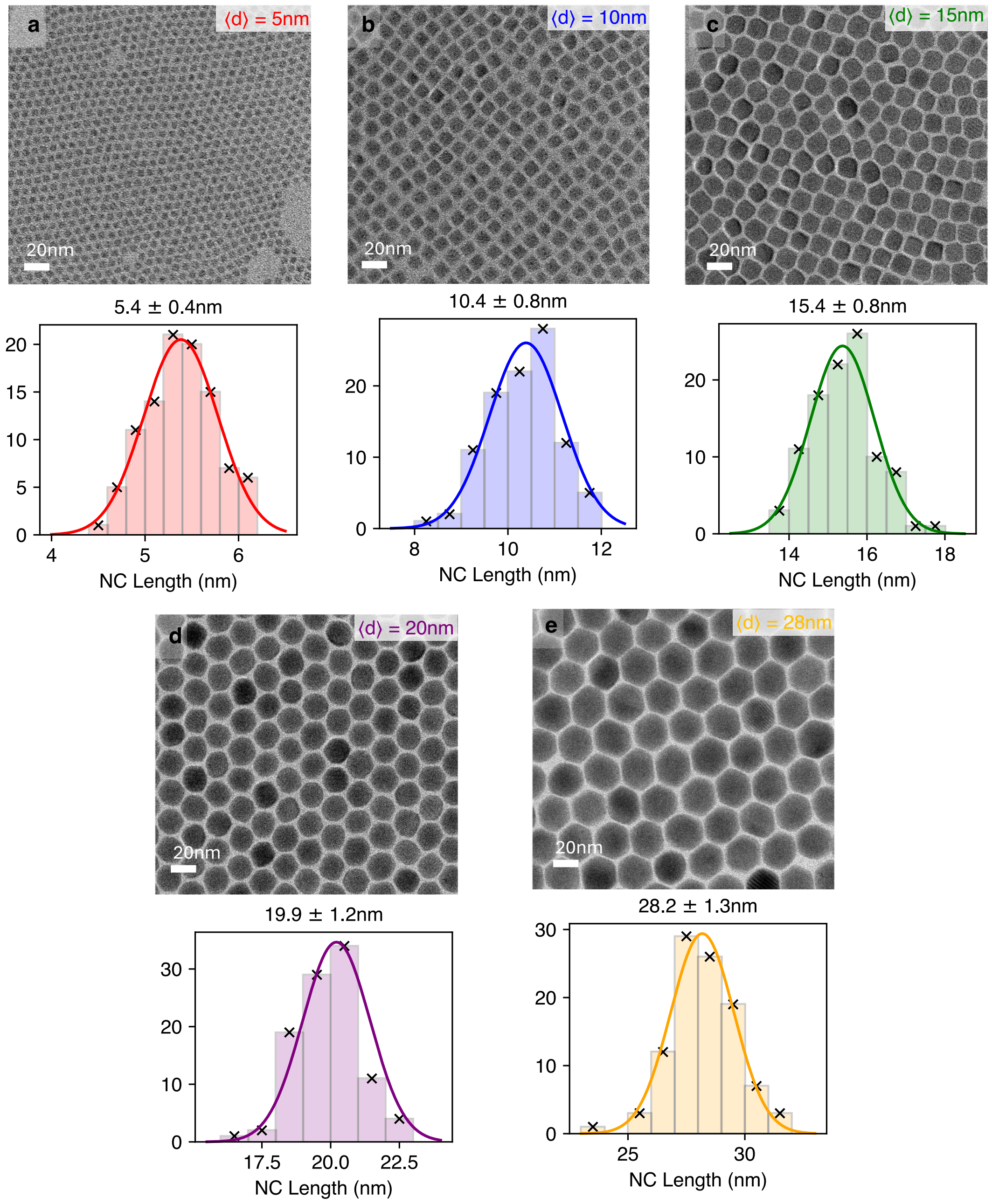

Figure S1 – Transmission electron microscopy micrographs of monolayer films of $CsPbBr_3$ nanocrystals with nominal edge lengths 5nm (a), 10nm (b), 15nm (c), 20nm (d) and 28nm (e). The edge length distribution of each sample is shown as a histogram, fit with a Gaussian function.

3.2 X-Ray Diffraction

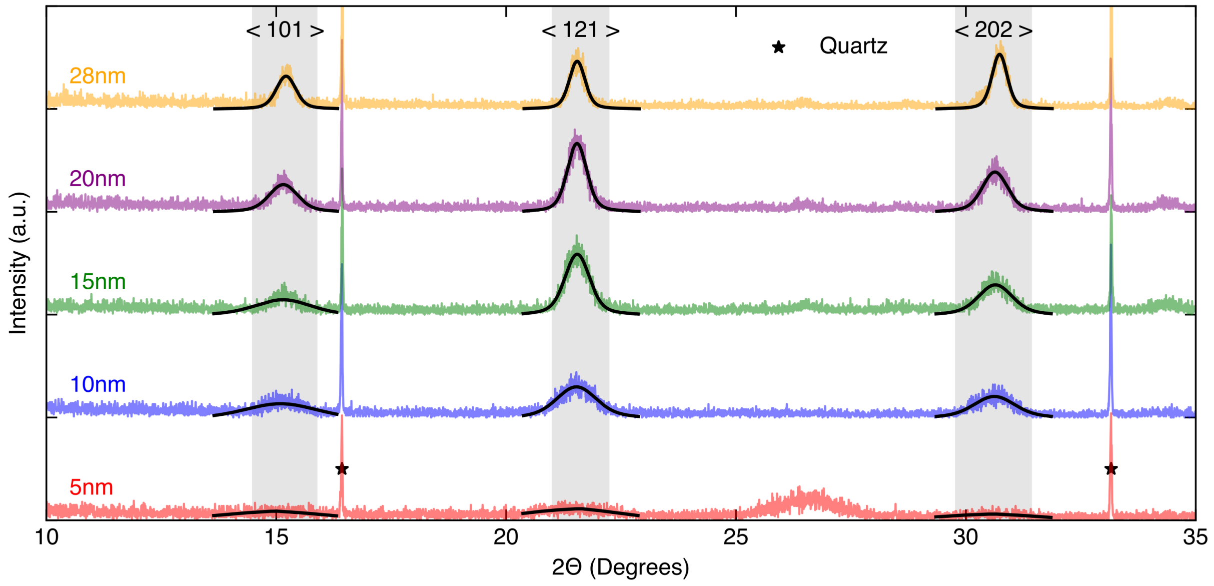

Figure S2 – X-ray diffraction (XRD) patterns of colloidal $CsPbBr_3$ nanocrystals of edge lengths 5, 10, 15, 20 and 28 nm with DOPE ligands, deposited as a thin film. Peaks associated with the quartz substrates are labelled with a star. The 16.43° z-cut quartz peak is used as a reference to correct the angles for sample tilt. Three perovskite peaks are identified (gray-colored regions) and fit with pseudo-Voigt functions. The peaks weaken in intensity and broaden with decreasing nanocrystal size. The XRD patterns are given an offset for visualization purposes.

4 Raman Analysis

4.1 Identifying Substrate Modes

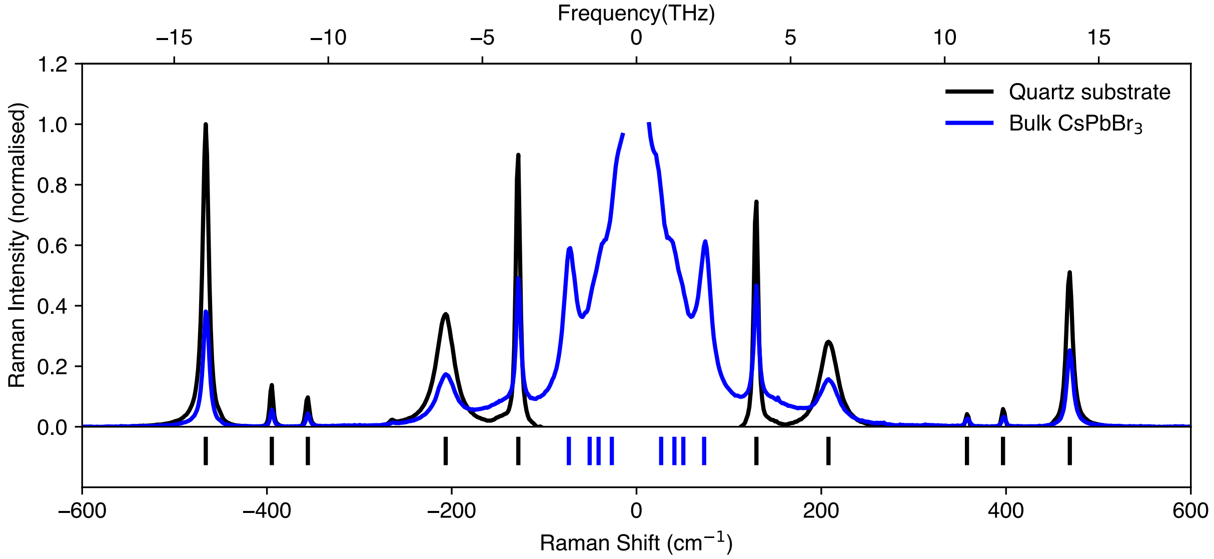

Figure S3 – Raman spectra of an 800nm-thick film of bulk $CsPbBr_3$ (blue) on quartz, and a bare quartz substrate (black). The central frequencies of the identified quartz and $CsPbBr_3$ peaks are shown below.

4.2 Modelling Nanocrystal Size-Dependent Raman Modes

The surface-area-to-volume ratio of individual nanocrystals scales inversely with nanocrystal edge length. Within the diffusion length of phonons, the probability of a surface interaction should thus scale inversely with nanocrystal diameter. Qualitatively therefore, the surface effects described in the main text must also become more dominant as the nanocrystal size decreases. Boundaries are inherently defective, providing rapid phonon decay mechanisms such as phonon-boundary scattering and surface strain.

We quantify this effect using Matthiessen's rule for the combined phonon lifetime[3], τ in a system with multiple decay pathways with lifetime τ_i

$$\tau^{-1} = \sum_i \tau_i^{-1} \tag{S2}$$

Equation S2 is often employed to decompose the different phonon scattering and broadening in metal halide perovskite materials[4–9]. Specific scattering mechanisms are phonon-phonon[3,10,11], phonon-boundary[9,12] and phonon-defect[8,13]. Here, we are interested in the phonon decay mechanisms induced by the nanocrystal surfaces. As the nanocrystals are of the same material and measured at the same temperature, the different sizes of nanocrystals will differ only in phonon-defect and phonon-boundary scattering rates. The combined phonon lifetime is modelled with independent contributions from intrinsic (bulk-like), τ_0, surface defect, τ_{SD}, and boundary-induced geometric localization, τ_{SL}

$$\tau^{-1} = \tau_0^{-1} + \tau_{\mathrm{SD}}^{-1} + \tau_{\mathrm{SL}}^{-1} \tag{S3}$$

τ_{SL} describes the physical constraints to phonon transport induced by the nanocrystal boundary. The phonon lifetime is modelled using the Casimir formalism of phonon transport, where the nanocrystal edge length is larger than the phonon wavelength, but on the same order of magnitude as the mean free path[14,15]. This is calculated from the group velocity, $\mathrm{v_g}$, and nanocrystal edge length, d as $\tau_{\mathrm{SL}}^{-1} = \mathrm{v_g}/\mathrm{d}$, assuming diffuse scattering. Defect scattering is modelled under a point defect formalism, in which $\tau_{\mathrm{SD}}^{-1} \propto \mathrm{n}\omega^4$, where n is the surface defect density[3]. Under simplified assumptions of the surface defect density scaling, n is proportional to the surface-area-to-volume ratio $n \propto d^2/d^3$, meaning that the defect scattering rate scales inversely with nanocrystal edge length. These two scattering rates are inversely proportional to the nanocrystal edge length, and as such can be combined into a single surface scattering term. Employing the uncertainty relation $\Gamma \sim \tau^{-1}$, we reproduce Equation 2 in the main text describing the Raman mode broadening parameter as a function of nanocrystal edge length, as follows:

$$\Gamma = \Gamma_0 + \frac{A}{d} \tag{S4}$$

where Γ_0 and A are fit parameters corresponding to the intrinsic broadening and a proportionality constant respectively. Given the frequency dependence of both τ_{SD} and τ_{SL}, a different A value is required for different Raman modes.

Similar $\Gamma \sim d^{-n}$ relationships have been reported analytically and empirically to explicitly quantify the surface-induced broadening mechanisms in traditional low-dimensional semiconductors[14,16–18]. The boundary-induced geometric localisation term in Equation S3 accounts for the classical effect of phonons interacting with a nanocrystal boundary within its mean free path length. This boundary effectively limits long-range phonon transport. Nanocrystalline materials on the much smaller length scale of the phonon wavelength are subject to phonon confinement. At this length scale, Raman selection rules are relaxed allowing off-centre ($q \neq 0$) phonons to become Raman active, inducing a similar size-dependent broadening of Raman modes, as well as a redshift with decreasing size[19,20]. This has previously been observed for long-wavelength acoustic phonons in quantum dots[21,22]. In this study, we consider optical phonons with much shorter wavelengths. The low phonon group velocities measured in metal halide perovskites ($\sim 10^3$ ms^{-1})[23–25] at THz frequencies yields an estimation of the phonon wavelength on the order of $\lambda <1$ nm. Nanocrystals of edge lengths 5-28 nm are much larger than the length scale of phonon confinement. Additionally, no size-dependent energy shift in the Raman modes is observed. As such, the size-dependent vibrational properties of the nanocrystals observed here are therefore dominated by phonon scattering processes at the surfaces, enabled by the large surface-area-to-volume ratio.

5 Ligand-dependent Characterization

5.1 Steady State Optical Spectroscopy

Figures S4 and S5 show the absorbance and photoluminescence spectra respectively, for 28 nm diameter $CsPbBr_3$ nanocrystals with each of the four ligands discussed in the main text. The magnitude of absorbance at the exciton peak (~2.42 eV) differs between films, because of the significant differences in thickness of the solution-processed films. Very small differences (~10meV) in the energies of the absorption onset are seen between different ligands. Such subtle differences have been reported in processing identical nanocrystal materials in different solvents, and so we attribute this observation to the different sample preparation methods discussed in Section S1. We note that the real edge lengths of the nanocrystals differ slightly between different ligands, as shown in the transmission electron microscopy analysis in Section S5.3. However, in such a weak confinement regime, 1-2nm edge length difference has a minimal influence on the absorption and emission energies. The photoluminescence (PL) spectra in Figure S5 exhibit more substantial differences, both in linewidth and peak energy. Broad, asymmetric PL spectra of colloidal nanocrystals are evidence of the size distribution within the ensemble on the film. Furthermore, PL redshift and asymmetric broadening can arise from photon reabsorption. High energy photons emitted via radiative recombination deep within the film can be reabsorbed before escaping the surface, and their energy redistributed[26,27]. Steady-state spectra cannot differentiate the photons that are reabsorbed and reemitted, resulting in an asymmetric redshifted spectrum[28]. Table S1 shows that the thin film thickness varies considerably between films of each ligand, leading to pronounced differences in photon reabsorption effects, as photons emitted in a thicker film have a higher probability of being reabsorbed. We attribute differences in PL to these two effects.

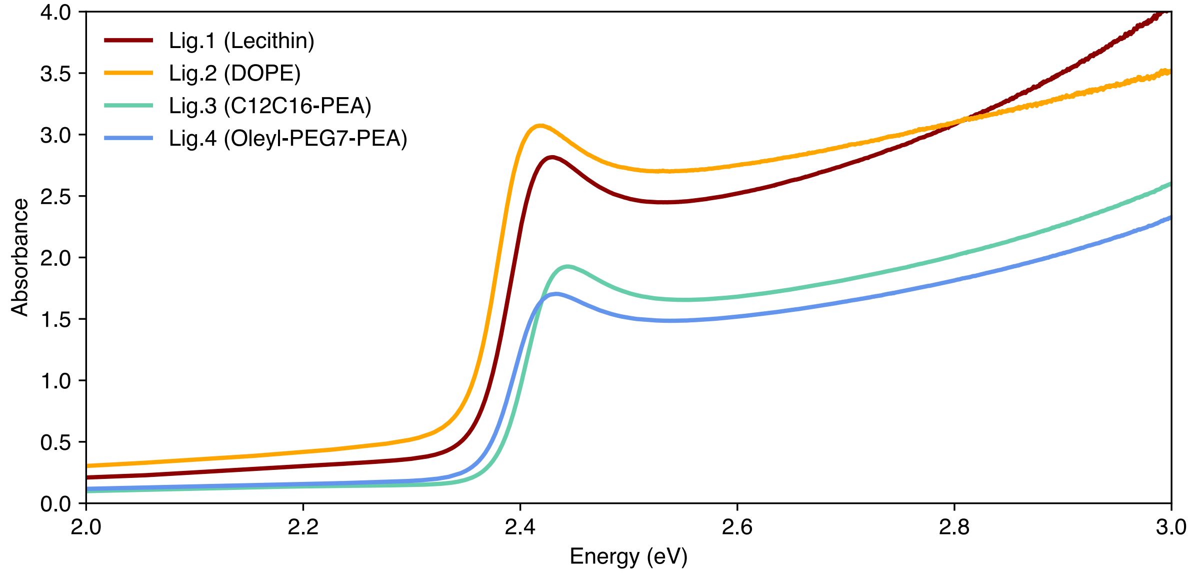

Figure S4 – Linear absorbance spectra of the 28 nm $CsPbBr_3$ nanocrystal films with four different ligands, Ligand 1: lecithin, Ligand 2: DOPE, Ligand 3: C12C16-PEA, Ligand 4: Oleyl-PEG7-PEA.

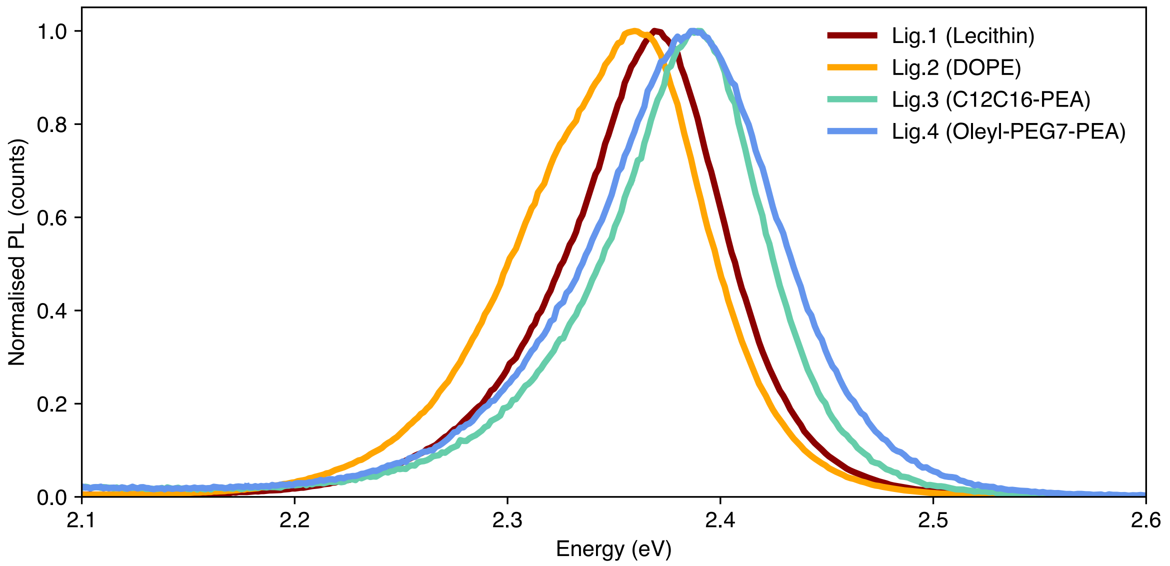

Figure S5 – Steady-state photoluminescence spectra of the 28nm $CsPbBr_3$ nanocrystal films with four different ligands.

5.3 Transmission Electron Microscopy

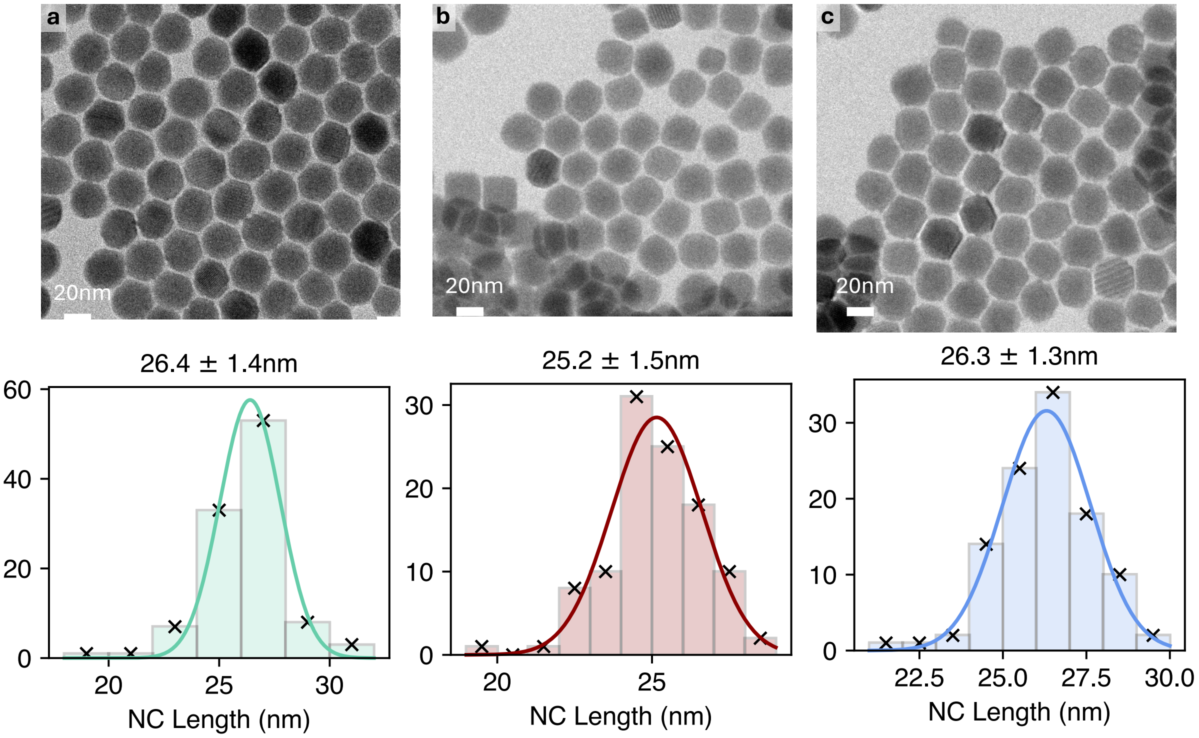

Figure S6 – Transmission electron microscope images of the nominally 28 nm $CsPbBr_3$ nanocrystals with ligands C12C16-PEA (a), Lecithin (b), Oleyl-PEG7-PEA (c). The 28 nm nanocrystals with DOPE are the same thin films as used in the size-dependent series, the TEM image can be found in Figure S1. The edge length distribution of each sample is shown as a histogram, fit with a Gaussian function.

5.4 X-Ray Diffraction

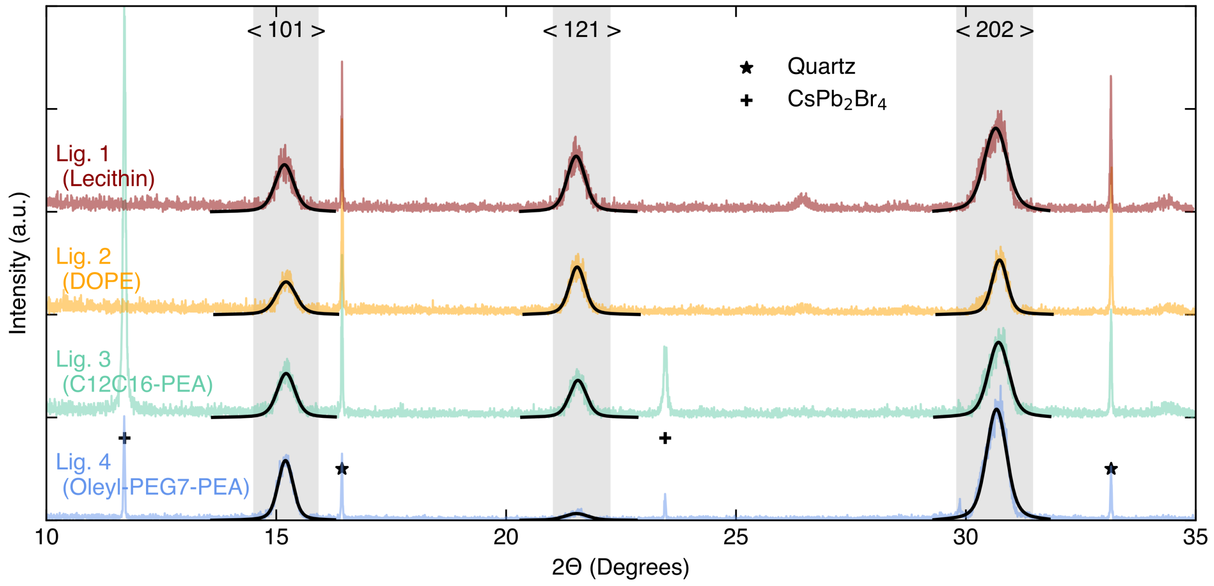

Figure S7 – X-ray diffraction (XRD) patterns of 28 nm colloidal $CsPbBr_3$ nanocrystals with four different ligands, deposited as thin films on quartz. Peaks associated with the quartz substrates are labelled with a star, and those corresponding to a secondary phase, $CsPb_2Br_5$, are labelled with a cross. The 16.43° z-cut quartz peak is used as a reference to correct the angles for sample tilt. Three perovskite peaks are identified (gray-colored regions) and fit with pseudo-Voigt functions. The amplitude in the XRD pattern of nanocrystals with Ligand 4 is scaled down for better comparison, and others are given an offset for visualization.

5.5 Film Thickness

The thickness of thin films used for all measurements except TEM was determined using a Dektak 150 surface profilometer. The films were scratched down to substrate level and the stylus scanned across the scratch. At least 10 measurements were performed across different sections of the film to achieve an average thickness.

	**Film Thickness (nm)**
Ligand 1 (Lecithin)	879 ± 109
Ligand 2 (DOPE)	273 ± 47
Ligand 3 (C12C16-PEA)	360 ± 31
Ligand 4 (Oleyl-PEG7-PEA)	416 ± 67

Table S1 – Thickness measurements of the thin films of 28 nm-edge length nanocrystals with different ligands deposited on z-cut quartz substrates using a Dektak profilometer.

5.6 Ligand-Dependent Linewidths of Raman Modes

Figure S8 shows the ligand-dependent Stokes and anti-Stokes Raman mode linewidths determined from the Lorentz oscillator model described in Equation 1. The identified mode 'M1' is also shown in Figure 4a of the main text. A similar systematic variation across ligand choice is observed in 3 of the identified modes. To investigate the robustness of our fitting method, we calculated the residuals of the model to our experimental data. Residuals were found to be random and normally distributed for all 4 of the measured samples, suggesting the model is statistically self-consistent, fully capturing the experimental data within Gaussian experimental noise.

To further test the quality of the model, and to produce uncertainties in the determination of model parameters, we performed a bootstrap analysis to the experimental data. Experimental noise, determined from the Raman response far from the observed modes (>10 THz), is used as an estimate of the experimental error. The experimental data is resampled within this experimental noise, and the fitting protocol of a least squares fit to Equation 1 is performed on this artificial dataset. We repeated this process 25 times and took the standard deviation of the parameter set as the error in extracted Raman mode linewidths.

We note that the 'central' mode M4 lacks an apparent ligand-dependent systematic variation in linewidth, as observed in Modes 1-3. The central mode in metal halide perovskites has been observed to be independent of extrinsic sources of disorder[29], and so may not be affected by the ligand surface chemistry.

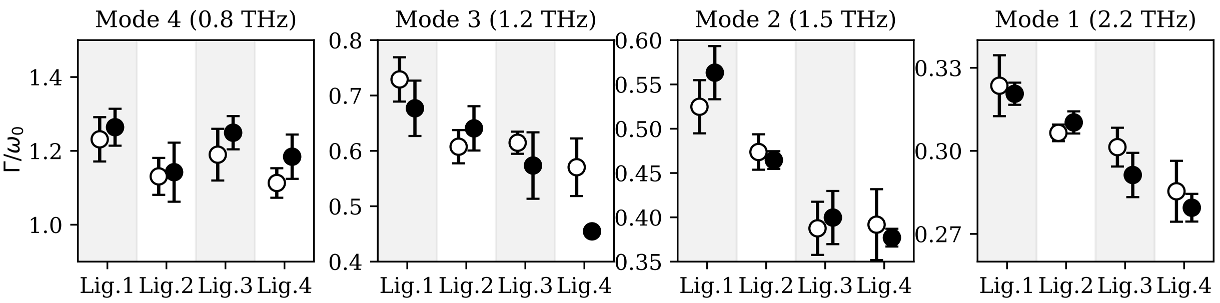

Figure S8 – Normalized Raman linewidths of the 4 identified Raman modes measured in thin films of 28 nm $CsPbBr_3$ nanocrystals with different ligands extracted from Raman spectra shown in Figure 3 of the main text, Ligand 1: lecithin, Ligand 2: DOPE, Ligand 3: C12C16-PEA, Ligand 4: Oleyl-PEG7-PEA. The linewidths of the most prominent mode, Mode 1, is shown in Figure 4 in the main text. No significant differences are observed in the central mode (Mode 4) with ligand choice. A consistent, systematic variation of the linewidth is observed in Modes 1-3. The Stokes Raman (open circles) and anti-Stokes Raman (filled circles) are shifted relative to one another to allow for easier visualisation.

6 Time-Resolved Spectroscopy

6.1 Time-Resolved Photoluminescence

We note that the size distribution of nanocrystals within the film generates an inhomogeneous energy landscape due to differing degrees of quantum confinement. The measured PL decay is a sum over an ensemble of different emitters. As such, a simple charge-carrier recombination model involving mono-exponential population decay of first order non-radiative, and first-order excitonic radiative recombination is not appropriate to describe the observed dynamics. Instead, the PL lifetime is determined from the time taken for the intensity to decay to 1/e of the initial peak value, a metric which is universally comparable across different films.

Photoluminescence lifetimes provided in Figure 4d are defined as the time taken for the number of PL counts to decay to 1/e of the peak value. Due to systematic error in the histograms of Figure S9, the PL lifetimes shown in Figure 4d are the average of points that fall within a 5% range of this 1/e criterion, including data for all 3 of the fluences measured. The uncertainty in lifetime is calculated as the standard deviation of these points.

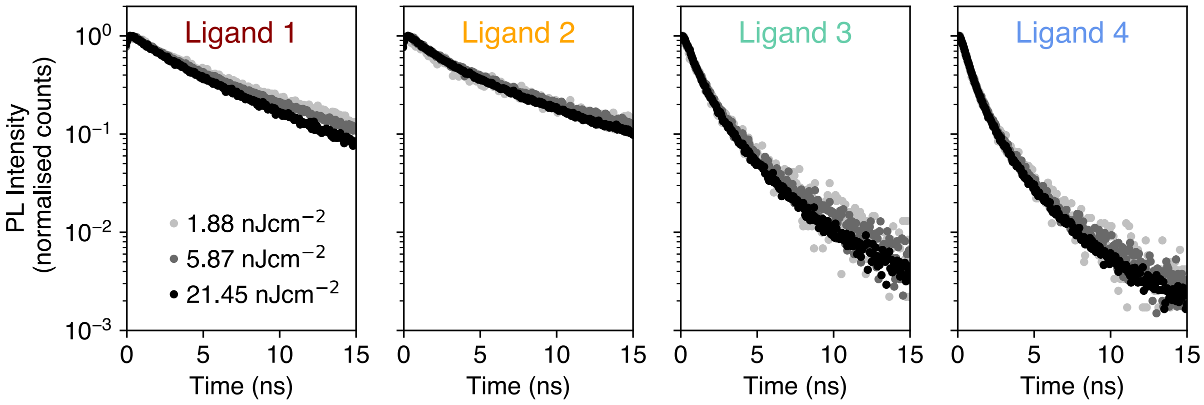

Figure S9 – Time-resolved photoluminescence transients of 28 nm $CsPbBr_3$ nanocrystals for each of the four ligands discussed in the main text, Ligand 1: lecithin, Ligand 2: DOPE, Ligand 3: C12C16-PEA, Ligand 4: Oleyl-PEG7-PEA. Each film was measured at the three different excitation fluences indicated in the legend to the left panel. The transients are normalized to highlight the similar dynamics observed at each fluence.

6.2 Fluence-Dependent Optical-Pump Terahertz Probe (OPTP) Spectroscopy

The effective sum electron-hole charge-carrier mobility is determined from the peak of photoconductivity, within the first 2ps in Figure S10. This metric is sensitive only to processes occurring on a similar timescale as thermalisation. As discussed in the main text, the sub-picosecond trapping of hot carriers generates free charges that contribute to the observed photoconductivity. Similarly, higher-order multiexciton complexes such as trions may form on this timescale, or indeed throughout the proceeding dynamics observed in Figure S10, as a free charge carrier generated via hot-carrier trapping forms a bound state with an exciton. Due to its charge, a trion would still contribute to the observed photoconductivity, though with a lower mobility. In large nanocrystals such as the 28 nm nanocrystals studied here, the trion binding energy has been observed to be much lower than the thermal energy, so the free charge carrier response dominates the photoconductivity response[30]. Qualitatively however, the observed systematic variation of charge-carrier mobility between nanocrystal films with different ligands is valid in the case of co-existing free carriers and charged multiexciton complexes.

The frequency region probed here (0.5-2.5 THz) is well below the frequency for excitonic resonances, given the high exciton binding energy in $CsPbBr_3$, in some reports as high as 40 meV for bulk $CsPbBr_3$, but likely pushed higher by confinement effects in nanocrystals[31,32]. IR-active phonon modes within this region can modulate the response, as observed by Motti et al.[33], though we expect negligible changes in phonon oscillator strength with ligand and so this effect on the frequency-averaged charge-carrier mobility determination should be low. We note that phonon modulation similarly has no effect on the observed shape of the conductivity transients in the sub-nanosecond regime, as reported by Motti et al.[33]

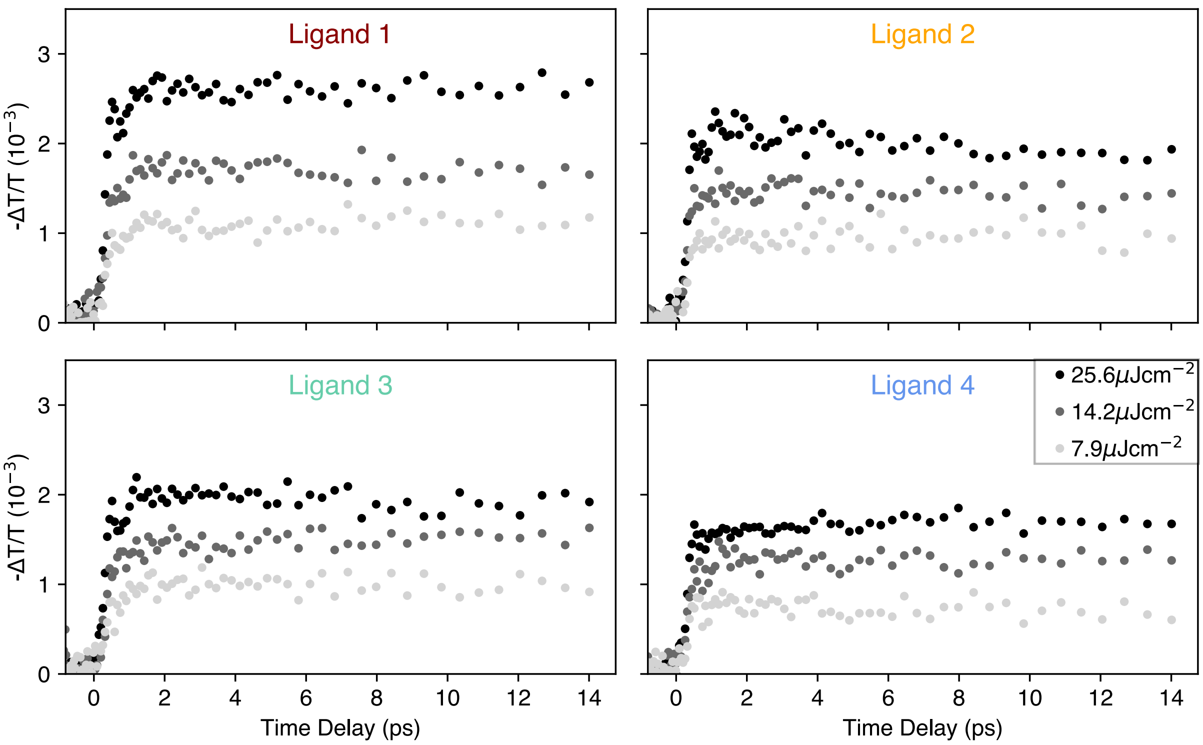

Figure S10 – OPTP transients measured for 28 nm $CsPbBr_3$ nanocrystals with each of the four ligands, Ligand 1: lecithin, Ligand 2: DOPE, Ligand 3: C12C16-PEA, Ligand 4: Oleyl-PEG7-PEA. The samples were photoexcited by 3.1 eV photons at the three excitation fluences indicated in the legend of the bottom right graph. Over the shown timescale covering the first 15ps following excitation, the decay dynamics are relatively flat. The effective electron-hole sum mobility is determined from the peak of photoconductivity within the first 2ps.

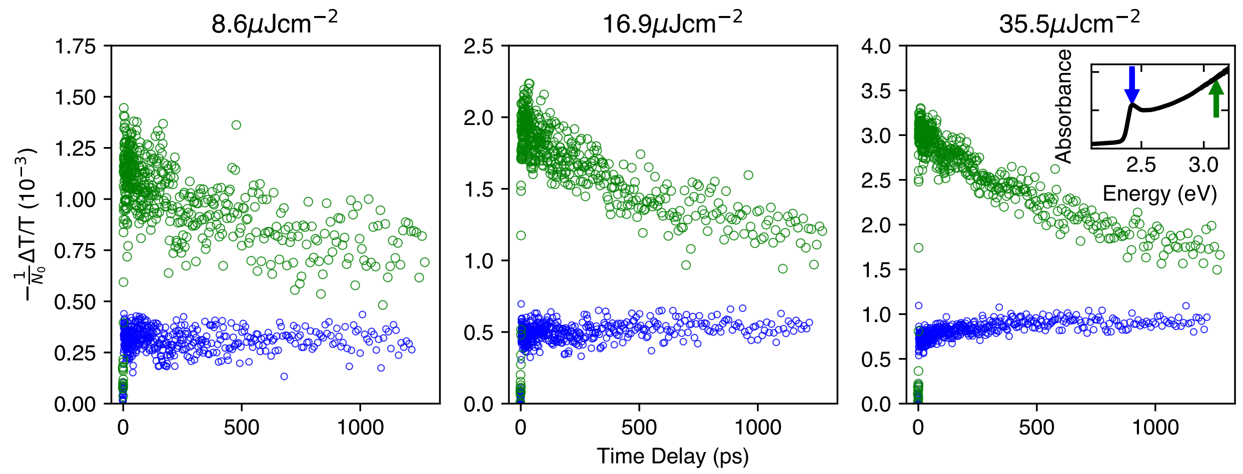

Figure S11 – OPTP transients of a thin film of 28 nm nanocrystals capped with lecithin (ligand 1), excited at 3.1 eV (green) and 2.4 eV (blue) at the three different fluences indicated above the figure. The transients are normalized by the initial charge carrier density upon photoexcitation. The inset shows the absorbance spectrum of Lecithin, highlighting the energetic location of the two pump beams. At all three fluences, excitation high in the band (3.1 eV) shows a higher peak and faster decay than excitation at the band edge (2.4 eV).

6.3 Calculating the Effective Charge-Carrier Mobility

The effective electron-hole sum mobility was calculated from OPTP measurements. The change in terahertz transmittance signal is directly proportional to the sheet photoconductivity in the film, expressed as

$$\Delta S = -\epsilon_0 c(n_A + n_B)\frac{\Delta T}{T} \tag{S5}$$

where ϵ_0 is the vacuum permittivity, c is the speed of light, and n_A, n_B are the real THz refractive indices of the materials interfacing the perovskite layer (vacuum and z-cut quartz respectively). $\Delta T/T$ is the photo-induced change in terahertz transmittance, evaluated at the peak immediately following photoexcitation relative to the transmittance without photoexcitation. The number of photoexcited free charge carriers, N, at this peak of photoconductivity is known:

$$N = \phi \frac{E\lambda}{hc}\left(1 - T_{pump} - R_{pump}\right) \quad (S6)$$

where ϕ is the branching ratio between absorbed photons and generated charge carriers, E is the incident pump energy per pulse, λ is the pump excitation wavelength, h is Planck's constant, and T_{pump}, R_{pump} are the fractional transmission and reflection of the sample as measured at the pump wavelength. The effective charge-carrier mobility is then calculated using these known parameters

$$\mu = \frac{\Delta S A_{eff}}{Ne} \quad (S7)$$

where e is the electron charge and A_{eff} is the effective area derived from the overlap of the pump and probe beams. Using Equations 5 and 6 yields the following expression for the effective charge-carrier mobility using measurable quantities

$$\phi\mu = -\frac{\epsilon_0 c(n_A + n_B)A_{eff}hc}{Ee\lambda(1 - T_{pump} - R_{pump})}\left(\frac{\Delta T}{T}\right) \quad (S8)$$

Here, μ is the electron-hole sum mobility, while ϕ is the ratio of generated free charge carriers to absorbed photons. Given that the THz probe is effectively "blind" to excitons, whose inter-excitonic transitions do not fall into the THz spectral range probed here, ϕ mostly represents the branching ratio of free charges to total generated photoexcitation (i.e. exciton and free-charge) densities. Effective charge-carrier mobilities given in Figure 4b are calculated using Equation S8 from photoconductivity transients in Figure S9. The peak $\Delta T/T$ was averaged over at least 10 measurements. The determined $\phi\mu$ values and their corresponding errors given in Figure 4b are averaged over measurements at the three fluences shown in Figure S10, weighted by the statistical error in $\Delta T/T$.

As previously observed by Motti et al.[33], $CsPbBr_3$ nanocrystals exhibit a non-zero imaginary component of the photoconductivity. The Drude-Smith model can yield a charge-carrier mobility

incorporating boundary backscattering observed in nanocrystals, however, such models tend to produce unphysical values of μ approaching infinity with an increasing backscattering parameter, in particular for the confined nature of charge carriers in nanocrystals. We avoid these pitfalls here by extracting $\phi\mu$ from the frequency-averaged time-resolved photoconductivity. Furthermore, as discussed by Ulatowski et al.[34] in the regime of a weak THz conductivity response, the impact of a non-zero imaginary photoconductivity component is negligible. We also note that the choice of spectral analysis has no effect on the shape of the conductivity transients here.

(1) Akkerman, Q. A.; Nguyen, T. P. T.; Boehme, S. C.; Montanarella, F.; Dirin, D. N.; Wechsler, P.; Beiglböck, F.; Rainò, G.; Erni, R.; Katan, C.; Even, J.; Kovalenko, M. V. Controlling the Nucleation and Growth Kinetics of Lead Halide Perovskite Quantum Dots. *Science* **2022**, *377* (6613), 1406–1412. https://doi.org/10.1126/science.abq3616.

(2) Morad, V.; Stelmakh, A.; Svyrydenko, M.; Feld, L. G.; Boehme, S. C.; Aebli, M.; Affolter, J.; Kaul, C. J.; Schrenker, N. J.; Bals, S.; Sahin, Y.; Dirin, D. N.; Cherniukh, I.; Raino, G.; Baumketner, A.; Kovalenko, M. V. Designer Phospholipid Capping Ligands for Soft Metal Halide Nanocrystals. *Nature* **2024**, *626* (7999), 542–548. https://doi.org/10.1038/s41586-023-06932-6.

(3) Jung, Y.; Lee, W.; Han, S.; Kim, B.-S.; Yoo, S.-J.; Jang, H. Thermal Transport Properties of Phonons in Halide Perovskites. *Adv. Mater.* **2023**, *35* (43), 2204872. https://doi.org/10.1002/adma.202204872.

(4) Wright, A. D.; Verdi, C.; Milot, R. L.; Eperon, G. E.; Pérez-Osorio, M. A.; Snaith, H. J.; Giustino, F.; Johnston, M. B.; Herz, L. M. Electron–Phonon Coupling in Hybrid Lead Halide Perovskites. *Nat Commun* **2016**, *7* (1), 11755.

(5) Saran, R.; Heuer-Jungemann, A.; Kanaras, A. G.; Curry, R. J. Giant Bandgap Renormalization and Exciton–Phonon Scattering in Perovskite Nanocrystals. *Adv. Opt. Mater.* **2017**, *5* (17), 1700231. https://doi.org/10.1002/adom.201700231.

(6) Yu, B.; Zhang, C.; Chen, L.; Huang, X.; Qin, Z.; Wang, X.; Xiao, M. Exciton Linewidth Broadening Induced by Exciton–Phonon Interactions in $CsPbBr_3$ Nanocrystals. *J. Chem. Phys.* **2021**, *154* (21), 214502. https://doi.org/10.1063/5.0051611.

(7) Cheng, O. H.-C.; Qiao, T.; Sheldon, M.; Son, D. H. Size- and Temperature-Dependent Photoluminescence Spectra of Strongly Confined $CsPbBr_3$ Quantum Dots. *Nanoscale* **2020**, *12* (24), 13113–13118. https://doi.org/10.1039/D0NR02711A.

(8) Hu, Y.; Xu, J.; Ruan, X.; Bao, H. Defect Scattering Can Lead to Enhanced Phonon Transport at Nanoscale. *Nat. Commun.* **2024**, *15* (1), 3304. https://doi.org/10.1038/s41467-024-47716-4.

(9) Yang, Z.; Yang, F. Scattering of Phonons by Edge Dislocation and Thermal Conductivity of Nanocrystalline Silicon. *Micro Nanostructures* **2023**, *180*, 207608. https://doi.org/10.1016/j.micrna.2023.207608.

(10) Granados del Águila, A.; Do, T. T. H.; Xing, J.; Jee, W. J.; Khurgin, J. B.; Xiong, Q. Efficient Up-Conversion Photoluminescence in All-Inorganic Lead Halide Perovskite Nanocrystals. *Nano Res.* **2020**, *13* (7), 1962–1969. https://doi.org/10.1007/s12274-020-2840-7.
(11) Zhang, B.; Klarbring, J.; Ji, F.; Simak, S. I.; Abrikosov, I. A.; Gao, F.; Rudko, G. Y.; Chen, W. M.; Buyanova, I. A. Lattice Dynamics and Electron–Phonon Coupling in Double Perovskite $Cs_2NaFeCl_6$. *J. Phys. Chem. C* **2023**, *127* (4), 1908–1916. https://doi.org/10.1021/acs.jpcc.2c07493.
(12) Anufriev, R.; Maire, J.; Nomura, M. Reduction of Thermal Conductivity by Surface Scattering of Phonons in Periodic Silicon Nanostructures. *Phys. Rev. B* **2016**, *93* (4), 045411. https://doi.org/10.1103/PhysRevB.93.045411.
(13) Fon, W.; Schwab, K. C.; Worlock, J. M.; Roukes, M. L. Phonon Scattering Mechanisms in Suspended Nanostructures from 4 to 40 K. *Phys. Rev. B* **2002**, *66* (4), 045302. https://doi.org/10.1103/PhysRevB.66.045302.
(14) Han, Y.; Dong, J.; Qin, G.; Hu, M. Phonon Transport in the Ground State of Two-Dimensional Silicon and Germanium. *RSC Adv.* **2016**, *6* (74), 69956–69965. https://doi.org/10.1039/C6RA14351B.
(15) Bourgeois, O.; Tainoff, D.; Tavakoli, A.; Liu, Y.; Blanc, C.; Boukhari, M.; Barski, A.; Hadji, E. Reduction of phonon mean free path: From low-temperature physics to room temperature applications in thermoelectricity. *Comptes Rendus Phys.* **2016**, *17* (10), 1154–1160. https://doi.org/10.1016/j.crhy.2016.08.008.
(16) Yashenkin, A. G.; Utesov, O. I.; Koniakhin, S. V. Bench Tests for Microscopic Theory of Raman Scattering in Powders of Disordered Nonpolar Crystals: Nanodiamonds and Si Nanoparticles. *J. Raman Spectrosc.* **2021**, *52* (11), 1847–1859. https://doi.org/10.1002/jrs.6242.
(17) Koniakhin, S. V.; Utesov, O. I.; Yashenkin, A. G. Lifetimes of Confined Optical Phonons and the Shape of a Raman Peak in Disordered Nanoparticles. II. Numerical Treatment. *Phys. Rev. B* **2020**, *102* (20), 205422. https://doi.org/10.1103/PhysRevB.102.205422.
(18) Utesov, O. I.; Koniakhin, S. V.; Yashenkin, A. G. Effects of Bond Disorder and Surface Amorphization on Optical Phonon Lifetimes and Raman Peak Shape in Crystalline Nanoparticles. *J. Phys. Chem. C* **2021**, *125* (33), 18444–18455. https://doi.org/10.1021/acs.jpcc.1c04007.
(19) Zhang, P.; Feng, Y.; Anthony, R.; Kortshagen, U.; Conibeer, G.; Huang, S. Size-Dependent Evolution of Phonon Confinement in Colloidal Si Nanoparticles. *J. Raman Spectrosc.* **2015**, *46* (11), 1110–1116. https://doi.org/10.1002/jrs.4727.
(20) Amaechi, I. C.; Ruediger, A.; Pignolet, A. Phonon Confinement and Particle Size Effect on the Low-Frequency Raman Mode of Aurivillius Phase $Bi_4Ti_3O_{12}$ Powders. *RSC Adv. 13* (8), 4917–4923. https://doi.org/10.1039/d2ra06297f.
(21) Harkort, C.; Kalitukha, I. V.; Kopteva, N. E.; Nestoklon, M. O.; Goupalov, S. V.; Saviot, L.; Kudlacik, D.; Yakovlev, D. R.; Kolobkova, E. V.; Kuznetsova, M. S.; Bayer, M. Confined Acoustic Phonons in $CsPbI_3$ Nanocrystals Explored by Resonant Raman Scattering on Excitons. *Nano Lett.* **2025**. https://doi.org/10.1021/acs.nanolett.5c03342.
(22) Lv, Y.; Yin, C.; Zhang, C.; Wang, X.; Yu, Z.-G.; Xiao, M. Exciton-Acoustic Phonon Coupling Revealed by Resonant Excitation of Single Perovskite Nanocrystals. *Nat. Commun.* **2021**, *12* (1), 2192. https://doi.org/10.1038/s41467-021-22486-5.

(23) Singh, R.; Balasubramanian, G. Impeding Phonon Transport through Superlattices of Organic–Inorganic Halide Perovskites. *RSC Adv.* **2017**, *7* (59), 37015–37020. https://doi.org/10.1039/C7RA06794A.

(24) Haque, M. A.; Kee, S.; Villalva, D. R.; Ong, W.; Baran, D. Halide Perovskites: Thermal Transport and Prospects for Thermoelectricity. *Adv. Sci.* **2020**, *7* (10), 1903389. https://doi.org/10.1002/advs.201903389.

(25) Handa, T.; Yamada, T.; Nagai, M.; Kanemitsu, Y. Phonon, Thermal, and Thermo-Optical Properties of Halide Perovskites. *Phys. Chem. Chem. Phys.* **2020**, *22* (45), 26069–26087. https://doi.org/10.1039/D0CP04426A.

(26) Du, J.; Righetto, M.; Kober-Czerny, M.; Yan, S.; Elmestekawy, K. A.; Snaith, H. J.; Johnston, M. B.; Herz, L. M. Inter-Layer Diffusion of Excitations in 2D Perovskites Revealed by Photoluminescence Reabsorption. *Adv. Funct. Mater.* **2025**, *35* (26), 2421817. https://doi.org/10.1002/adfm.202421817.

(27) Crothers, T. W.; Milot, R. L.; Patel, J. B.; Parrott, E. S.; Schlipf, J.; Müller-Buschbaum, P.; Johnston, M. B.; Herz, L. M. Photon Reabsorption Masks Intrinsic Bimolecular Charge-Carrier Recombination in $CH_3NH_3PbI_3$ Perovskite. *Nano Lett* **2017**, *17* (9), 5782–5789.

(28) Diab, H.; Arnold, C.; Lédée, F.; Trippé-Allard, G.; Delport, G.; Vilar, C.; Bretenaker, F.; Barjon, J.; Lauret, J.-S.; Deleporte, E.; Garrot, D. Impact of Reabsorption on the Emission Spectra and Recombination Dynamics of Hybrid Perovskite Single Crystals. *J. Phys. Chem. Lett.* **2017**, *8* (13), 2977–2983. https://doi.org/10.1021/acs.jpclett.7b00998.

(29) Lim, V. J.-Y.; Righetto, M.; Yan, S.; Patel, J. B.; Siday, T.; Putland, B.; McCall, K. M.; Sirtl, M. T.; Kominko, Y.; Peng, J.; Lin, Q.; Bein, T.; Kovalenko, M.; Snaith, H. J.; Johnston, M. B.; Herz, L. M. Contrasting Ultra-Low Frequency Raman and Infrared Modes in Emerging Metal Halides for Photovoltaics. *ACS Energy Lett.* **2024**, *9* (8), 4127–4135. https://doi.org/10.1021/acsenergylett.4c01473.

(30) Zhu, C.; Nguyen, T.; Boehme, S. C.; Moskalenko, A.; Dirin, D. N.; Bodnarchuk, M. I.; Katan, C.; Even, J.; Rainò, G.; Kovalenko, M. V. Many-Body Correlations and Exciton Complexes in $CsPbBr_3$ Quantum Dots. *Adv. Mater.* **2023**, *35* (9), 2208354.

(31) Ramírez, D.; Riveros, G.; Díaz, P.; Verdugo, J.; Núñez, G.; Lizama, S.; Lazo, P.; Dalchiele, E. A.; Gau, D. L.; Marotti, R. E.; Anta, J. A.; Contreras-Bernal, L.; Riquelme, A.; Idigoras, J. Electrochemically Assisted Growth of $CsPbBr_3$-Based Solar Cells Without Selective Contacts. *ChemElectroChem* **2020**, *7* (19), 3961–3968.

(32) Fröhlich, D.; Heidrich, K.; Künzel, H.; Trendel, G.; Treusch, J. Cesium-Trihalogen-Plumbates a New Class of Ionic Semiconductors. *J. Lumin.* **1979**, *18–19*, 385–388. https://doi.org/10.1016/0022-2313(79)90146-7.

(33) Motti, S. G.; Krieg, F.; Ramadan, A. J.; Patel, J. B.; Snaith, H. J.; Kovalenko, M. V.; Johnston, M. B.; Herz, L. M. $CsPbBr_3$ Nanocrystal Films: Deviations from Bulk Vibrational and Optoelectronic Properties. *Adv. Funct. Mater.* **2020**, *30* (19), 1909904. https://doi.org/10.1002/adfm.201909904.

(34) Ulatowski, A. M.; Herz, L. M.; Johnston, M. B. Terahertz Conductivity Analysis for Highly Doped Thin-Film Semiconductors. *J. Infrared Millim. Terahertz Waves* **2020**, *41* (12), 1431–1449. https://doi.org/10.1007/s10762-020-00739-6.
